# Chronic acidosis in the tumour microenvironment selects for overexpression of LAMP2 in the plasma membrane

**DOI:** 10.1038/ncomms9752

**Published:** 2015-12-10

**Authors:** Mehdi Damaghi, Narges K. Tafreshi, Mark C. Lloyd, Robert Sprung, Veronica Estrella, Jonathan W. Wojtkowiak, David L. Morse, John M. Koomen, Marilyn M. Bui, Robert A Gatenby, Robert J Gillies

**Affiliations:** 1 Department of Cancer Imaging and Metabolism, Moffitt Cancer Center and Research Institute, 12902 Magnolia Drive, Tampa, Florida 33612, USA; 2 Analytic Microscopy Core, Moffitt Cancer Center and Research Institute, 12902 Magnolia Drive, Tampa, Florida 33612, USA; 3 Department of Proteomics, Moffitt Cancer Center and Research Institute, 12902 Magnolia Drive, Tampa, Florida 33612, USA; 4 Department of Anatomic Pathology, Moffitt Cancer Center and Research Institute, 12902 Magnolia Drive, Tampa, Florida 33612, USA; 5 Department of Molecular Oncology, Moffitt Cancer Center and Research Institute, 12902 Magnolia Drive, Tampa, Florida 33612, USA

## Abstract

Early cancers are avascular and hence, profoundly acidic. Pre-malignant cells must adapt to acidosis to thrive in this hostile microenvironment. Here, we investigate MCF-7 cells that are adapted to grow in acidic conditions using SILAC proteomics and we reveal a significant upregulation of lysosomal proteins. Prominent among these is LAMP2 that functions to protect lysosomal membranes from acid proteolysis. LAMP2 upregulation by acidosis is confirmed both *in vitro* and *in vivo*. Furthermore, we show that the depletion of LAMP2 is sufficient to increase acidosis-mediated toxicity. In breast cancer patient samples, there is a high correlation of LAMP2 mRNA and protein expression with progression. We also observe that LAMP2 is located at the plasma membrane in clinical samples and this redistribution is acid-induced *in vitro*. Our findings suggest a potential adaptive mechanism, wherein cells chronically exposed to an acidic environment translocate lysosomal proteins to their surface, thus protecting the plasmalemma from acid-induced hydrolysis.

## Abstract (web summary)

The protein LAMP2 functions to protect lysosomal membranes from acid proteolysis. In this study, the authors show that malignant cells adapt to the acidic tumour microenvironment by redirecting LAMP2 from lysosomes to the plasma membrane.

## Graphical abstract

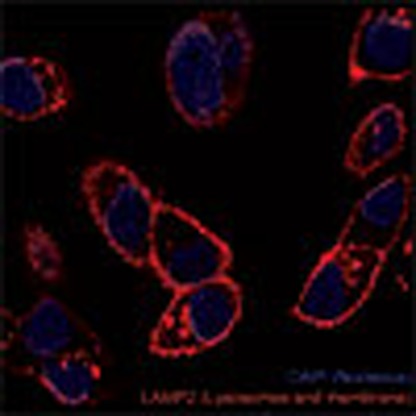

The acidic microenvironment of tumours strongly influences cancer progression and evolution^1^,^2^. A combination of poor vasculature perfusion, regional or intermittent hypoxia, and increased flux of carbons through fermentative glycolysis leads to extracellular acidosis in solid tumours, with extracellular pH values as low as 6.5 (ref. 3). Notably, environmental acidification likely occurs early in cancers, during the avascular phase of carcinoma *in situ* (CIS)^4^, wherein the nascent cancer cells undergo a metabolic switch towards a more glycolytic phenotype^5^, causing the interior of the lumen to become highly acidic. Notably, the glycolytic phenotype can become ‘hardwired' as Warburg proposed, leading to the continued generation of metabolic acids, even in well-oxygenated conditions. This acidified habitat imparts a Darwinian selection pressure that favours cells that have adapted mechanisms to be resistant to acid-mediated apoptosis. Furthermore, this phenotype is retained as cancers become locally invasive, a process known as ‘niche engineering', as solid tumours are unequivocally acidic (Fig. 1). An acidic microenvironment is thought to promote tumour progression, as acidosis can stimulate invasion and metastasis^1^,^5^,^6^,^7^, can be toxic to normal cells and mediate degradation and remodelling of the extracellular matrix (ECM)^8^, can elevate angiogenesis through the release of vascular endothelial growth factor^9^ and can inhibit the immune response to tumour antigens^10^. Cancer cells that are adapted to this chronic acidosis will survive and will have an advantage over non-adapted cells, such as normal epithelial and stroma cells. However, the survival mechanisms in the face of chronic acidosis are not well understood. Identifying these and defining a biomarker for acid-adapted cells would give some insight into tumour progression and potentially introduce novel imaging, diagnostic or therapeutic strategies.

To investigate mechanisms involved in acid adaptation, we initiated this work with a discovery phase, wherein we used whole-proteome screening to compare MCF-7 cells that were grown at neutral pH (acid naive or Non-adapted (NA)) with those that were grown at pH 6.7 until their growth rates matched those of the controls, ∼2 months (acid adapted (AA)). These studies revealed that lysosomal proteins were disproportionally increased in acid-adapted cells. This is of particular interest, because the rate of lysosomal–endosomal recycling is reported to be induced by acidic conditions^11^,^12^,^13^. Further, lysosomal enzymes, such as cathepsins, can be released by cells and play an important role in ECM remodelling^14^,^15^,^16^ and can stimulate angiogenesis, tumour growth and invasion^17^,^18^. Among acid-induced lysosomal proteins, lysosome-associated membrane protein-2 (LAMP2) is of particular interest because it is associated with chaperone-mediated autophagy, which is associated with acid adaptation^19^, and it is heavily glycosylated^20^, which can be predicted to provide an unstirred layer at the membrane surface to protect it from the low pH of lysosomes^20^,^21^. Validation studies confirmed LAMP2 expression upregulated by acidosis by reverse transcription–PCR and western blotting in MCF-7 and other breast cancer cell lines.

Our current study shows that LAMP2 is associated with acidic environments *in vitro*, *in vivo* and in patient samples. Studies with dorsal window chambers (DWCs) show that areas of high LAMP2 expression co-registered with areas of acidosis. Further, treatment of mice bearing breast cancer xenografts with bicarbonate buffer therapy to raise tumour pH, resulted in lower-LAMP2 expression compared with untreated controls. Immunohistochemistry (IHC) of human breast cancer tissue microarrays (TMAs) shows increased expression of LAMP2 with increasing tumour grade. Notably, high-resolution analyses of IHC samples showed that a significant amount of LAMP2 was expressed at the plasma membrane (PM), and this was confirmed by immunocytochemistry (ICC) of acid-adapted cells. Further, we show that cells expressing LAMP2 are more resistant to extreme acid-induced cytotoxicity, and knockdown of LAMP2 reverses this resistance. Further, acid-adapted cells formed well-defined spheroids, and this phenotype was reversed with knockdown of LAMP2. *In vivo*, short hairpin RNA (shRNA) knockdown of LAMP2 in acid-adapted cells induced a lag period before commencement of tumour growth. Hence, we propose a novel defence mechanism, wherein LAMP2 is translocated from lysosomes to the PM to protect the membrane from non-enzymatic or enzymatic acid hydrolysis.

## Results

### SILAC-proteomics biomarker discovery

**Figure 2:**
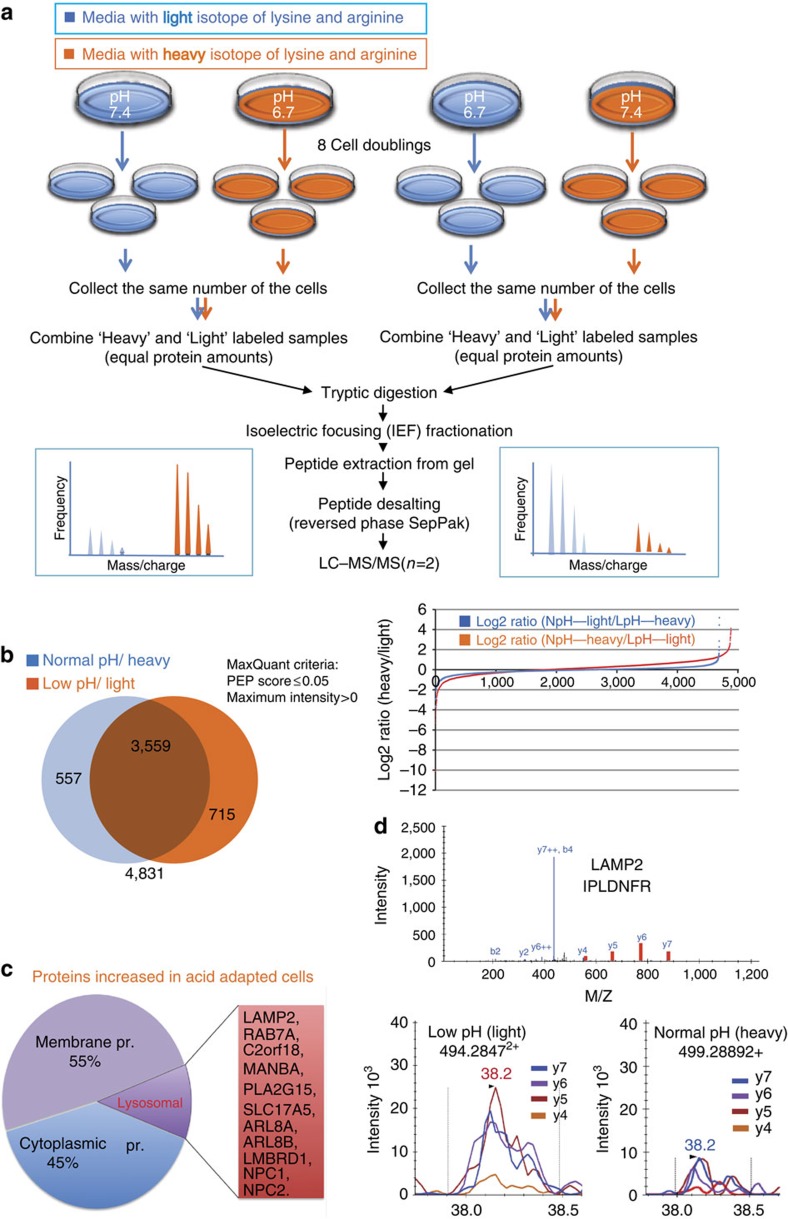
Shotgun SILAC-proteomics discovery approach. (**a**) Schematic of stable isotope labelling with amino acid in cell culture for MS analysis (SILAC-MS/MS). Acid-adapted and naive MCF-7 cells were grown either in media of normal isotopic distribution or in heavy (Δ6-Lys, Δ 10-Arg) medium for eight doublings (1 week). The cells were collected and lysed and combined for equal protein loads between the two groups. A label-flipping approach was employed through a parallel experiment, allowing for confident quantification and reducing false-positive biomarker associations. (**b**) The Venn diagrams show the number of protein discovered in each flipping experiment. The number underneath the diagram is the total protein number. Orange is the number of proteins with heavy label and blue with the light label. The graph beside the Venn diagram is observed log2 peak area ratios for flipping experiments plotted on the same graph. Candidate biomarkers were selected among proteins showing at least a 1.5-fold increase in expression under acidic conditions in both replicates across flipping experiments. (**c**) A Pie chart of proteins with increased expression in acid-adapted cells. In all, 45% of the proteins are cytoplasmic and 55% membrane proteins of which 12.8% are lysosomal and endosome proteins. (**d**) LC-MRM validation of discovered low-pH-induced proteins. MS/MS spectra of peptides from LAMP2 shown here, was extracted from our shotgun data set and used for the development of targeted multiple reaction monitoring (MRM) assays (optimized transitions indicated in red). Bottom panel: relative quantification was accomplished by comparing peak areas using Skyline.

In general, there have been numerous genomic- and transcriptomic-based studies to identify biomarkers for diagnosis and therapy prediction of cancers^22^. Proteomic studies are higher in actionable information content, as many mechanisms of biological control are exerted post-translationally and hence, proteomic approaches are highly suited for biomarker discovery. To analyse the effects of chronic acidosis on the proteome of cancer cells, we performed quantitative mass spectrometry (MS) on acid-adapted and acid-naive MCF-7 cancer cell lines metabolically labelled using stable isotope-labelled amino acids in cell culture (SILAC)^23^,^24^. Cells were considered acid-adapted following growth in low-pH culture conditions until their growth rates matched that of the control counterparts (pH 7.4) (Supplementary Fig. 1). To minimize the rate of false-positive biomarker association, parallel experiments were conducted in which the acid-adapted or acid-naive cells were labelled by growing them in SILAC ‘heavy' media (^13^C_6_ lysine and ^13^C_6_^14^N_4_ arginine), while the comparator cells (acid-naive or acid-adapted cells, respectively) were cultured in media containing the corresponding amino acids of naturally occuring isotopic distribution. The labelling strategy was reversed (flipped) to eliminate potential bias due to the media and incorporation of the stable isotope-labelled amino acids (Fig. 2a)^25^.

In total, 4,831 proteins were identified as consistent across the both SILAC experiments. MaxQuant software was used to determine the relative abundance of proteins between treatment conditions. To select potential biomarker candidates, we applied a cutoff of a 1.5-fold-higher expression under low-pH conditions that was consistently observed across label-flipping experiments. One hundred and four proteins met these criteria (Fig. 2b; Supplementary Table 1 shows the top 30 hits). Using the web-based DAVID functional classification tool^26^, this list was found to be significantly enriched for proteins associated with lysosomes, suggesting a potential role for this organelle in adaptation to the acidic environment (Fig. 2c; Supplementary Fig. 2). In all, 14 lysosomal proteins were thus identified as upregulated following acid adaptation: ARL8A, ARL8B, LMBRD1, NPC1, NPC2, RAB7A, C2orf18, LAMP1, LAMP2, MANBA, PLA2G15, SLC17A5, TMEM192 and TMEM55B.

### LC-MRM confirmation of candidates

To verify overexpression of biomarker candidates in the acid-adapted cells, protein candidates with more than 2-fold change (Supplementary Table 1) were selected for further validation by targeted liquid chromatography-multiple reaction monitoring (LC-MRM) MS^27^. Peptides were selected based on their observation in the discovery set (http://proteome.moffitt.org/QUAD/)^28^,^29^. Among the top 30 proteins in the discovery phase, both LAMP2 and S100A6 were very amenable to MRM analysis, and its quantification with LC-MRM was significantly different in acid-adapted and acid-naive cells (Fig. 2d).

### *In vitro* confirmation of discovered biomarkers

**Figure 3:**
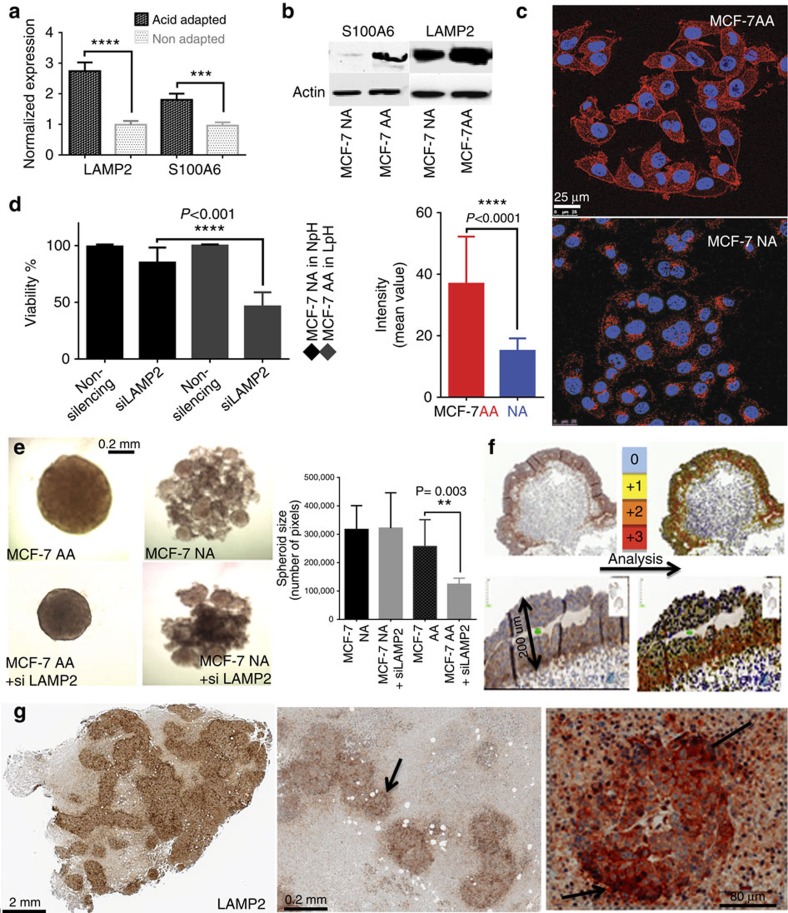
*In vitro* confirmation of proteomics results. (**a**) Quantitative reverse transcription–PCR and (**b**) western blot and (**c**) ICC results confirmed the higher expression level of LAMP2 in AA cells relative to NA MCF-7 cells. (**d**) LAMP2 siRNA treatment of NA and AA MCF-7 cells decreased the viability of acid-adapted cells much more than non-adapted cells. (**e**) siRNA treatment of spheres of AA and NA MCF-7 cells revealed that growth and size of AA spheres are affected by LAMP2 expression. The graph shows the average pixel count of spheres from both groups with error bars as s.d. (**f**) Representative images of spheroid 3D culture model (Rotary system) of MCF-7 cells showing the increased expression of LAMP2 close to the central part of the tumours at the oxygen diffusion limit (∼200 μm). (**g**) Representative images of heterogeneous LAMP2 expression (black arrow in IHC analysis) indicate high expression of LAMP2 at the suspected acidic regions of mouse tumour xenografts, such as the necrotic area neighbourhood and invasive edges.

To confirm proteomics results, we then applied quantitative reverse transcription–PCR, western blots and ICC to validate increased expression of LAMP2 and S100A6 in acid-adapted compared with acid-naive MCF-7 cell lines (Fig. 3a–c). Using these assays, expression of LAMP2 and S100A6 messenger RNA (mRNA) and protein were confirmed as being increased in acid-adapted versus acid-naive cells. To determine whether results were cell-line specific, validation experiments were also conducted in control and acid-adapted MCF-10-DCIS, ZR-75.1 and MDA-MB-231 breast cancer cell lines. Western blots of acid-adapted versus acid-naive cells showed overexpression of LAMP2 and S100A6 in all acid-adapted cells (Supplementary Fig. 3A,B).

To examine the role of LAMP2 on acid adaptation, NA and AA MCF-7 cells were treated with LAMP2 short interfering RNA (siRNA) (Fig. 3d; Supplementary Fig. 3C). These data showed that the AA MCF-7 were much more sensitive to siRNA knockdown, compared with NA cells, suggesting that they require LAMP2 for their survival much more than NA MCF-7 (also see below). To better understand the functional importance of LAMP2 on acidosis resistance, we studied the spheroid growth and clonogenicity of AA MCF-7 cells compared with NA MCF-7 using LAMP2 siRNA. In a clonogenic assay, knocking down LAMP2 lowered both the number of clones and the size of the clones in acidic pH, compared with untreated AA cells. The effect of knockdown is most marked in acid-adapted cells under acidic pH, and the effects were much larger and significant for the AA cells (Supplementary Fig. 3D,E). Spheroid assays were performed using hanging-droplet plates and non-adherent U-bottom 96-well plates. We grew spheroids of NA and AA MCF-7 cells at acidic and neutral pH with and without LAMP2 siRNA treatments. Notably, we consistently observed that the NA MCF-7 cells generated multiple clusters of small spheroids, whereas AA cells, whether LAMP2 downregulated or not, formed large well-formed spheroids in each well. Results showed that treatment with siRNA had little effect on the NA ‘spheroids', whereas it significantly reduced the volumes of AA spheroids (Fig. 3e).

### LAMP2 as an acidity biomarker in spheroids and xenografts

To simulate *in vivo* conditions, we also cultured MCF-7 cell line as three-dimensional (3D) spheroids using a zero-gravity Rotary Cell Culture System (3D). Because the oxygen diffusion limit in tissues is 0.16–0.2 mm, the cores of spheroids that are 1 mm in diameter and regions of tumours more than 0.2 mm from a blood vessel are more hypoxic and glycolytic and thus are expected to be acidic^30^. Beyond this penumbral region, the tissues are necrotic. Thus, we expect that acidity markers would exhibit regional expression at some distance (>0.1 mm) from the spheroid surface and close to the necrotic areas in xenografts. IHC staining of cancer cell spheroids showed overexpression of LAMP2 at the approximate oxygen diffusion limit (Fig. 3f; Supplementary Fig. 4A). The same spheroids were stained with S100A6. Notably, S100A6 was homogeneously expressed throughout the spheroid (Supplementary Fig. 4B). Spheroids of *p*-glycoprotein doxorubicin-resistant MCF-7 cells (MCF-DOX) and HCT-116 colon cancer cells were also stained with LAMP2 and we obtained the same results (Supplementary Fig. 4C). As controls, we also stained the same spheroids with GLUT1 as a positive marker for hypoxic/glycolytic cells. GLUT1 expression pattern was similar to that of LAMP2 (Supplementary Fig. 4D). We also stained breast tumour xenografts from Severe Combined Immune-Deficiency (SCID) mice (immunodeficient mice that have impaired ability of making T or B lymphocytes) for LAMP2 and S100A6. High LAMP2 staining was seen at the interface of necrotic areas with areas of fully viable cells and invasive edges of tumours (Fig. 3g; Supplementary Fig. 4E). In other studies, these areas have been shown to be acidic^8^. In both spheroids and tumour xenografts, S100A6 protein stained homogeneously (Supplementary Fig. 4B,E), in contrast to the acid-induced behaviour observed *in vitro* during our discovery phase. These observations suggest that, while S100A6 expression may be induced by low pH, there appear to be other factors that override the acid signal to regulate its expression *in vivo*.

### Tumours in DWC show expression of LAMP2 in acidic regions

**Figure 4:**
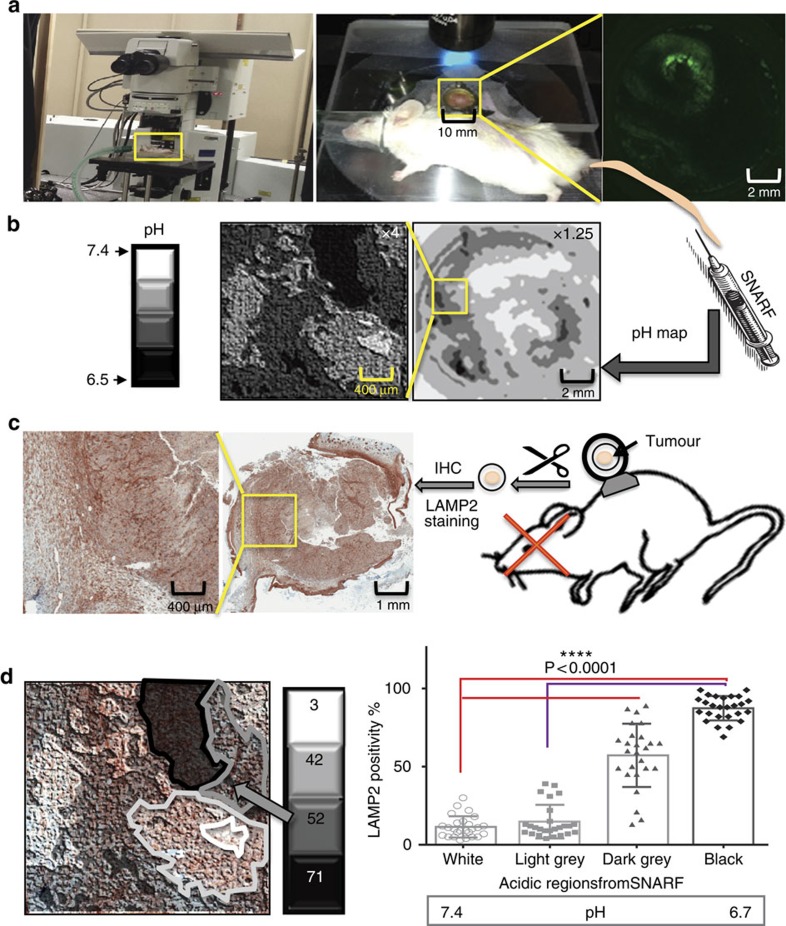
Correlation of marker expression with tumour pH using intravital imaging of SNARF-1 in DWC followed by IHC staining. (**a**) Regional extracellular tumour pH was determined by intravital imaging of the pH-sensitive fluorescent dye, SNARF-1, in breast cancer DWC xenograft tumours using an olympus multiphoton microscope. (**b**) Representative pH maps generated by the SNARF-1 ratiometric analysis on day 16 post tumour inoculation. Acidity ranges from low pH (black) to normal pH (white), meaning black is the most acidic area and white the least. (**c**) After the final SNARF-1 imaging, the tumours were extracted and fixed in the same orientation they were imaged and IHC stained for LAMP2 expression. The two left panels represent images of marker IHC staining for LAMP2 in two magnifications. (**d**) IHC staining images superimposed over the corresponding pH map (white, light grey, dark grey and black) to correlate the acidity of each region to expression of LAMP2 in the corresponding area. The stained tumours in each mapped region for acidity were analysed using positive pixel analysis by our custom-trained software in Definiene. The number in each box represents the percentage of cells that are LAMP2 positive. The correlation analysis showed significantly higher expression of LAMP2 in acidic areas of the tumours compared with non-acidic regions (Student's *t*-test, *P*<0.001 and error bars represent s.d.).

The DWC was developed in 1987 as an accurate tool to microscopically interrogate tumours and their microenvironment^31^ and we have adapted this system to measure intra- and peri-tumoral pH^8^,^32^. For DWC studies, we prepared spheroids of MCF-7, ZR-75.1 or MCF-7/GFP breast cancer cells, which were then implanted into the DWC and allowed to grow (Fig. 4a). Intravital microscopy was used to create a pH map using SNARF-1 (Seminaphtolrhodafluor-1, Life Technologies), a ratiometric pH-sensitive fluorescent probe that has altered emission wavelengths at basic and acidic pH values (Fig. 4b). Because of signal averaging and spatial kernelling, the ratiometric pH values were binned into four values, very low (<6.7; black), moderately low (6.7–6.95; dark grey), neutral (6.85–7.1; light grey) and moderately high (>7.1; white). Thereafter, tumours were harvested and fixed for histology. The margins of the tumours inside the chamber were marked to ensure proper registration of histology with *in vivo* images. The collected tumours were stained with LAMP2 antibody (Fig. 4c). Superposition of the acidity map from SNARF-1 with IHC staining was then used to correlate the expression level LAMP2 in different regions of the tumour with different pH_e_ values (Fig. 4d). These analyses showed that LAMP2 expression was significantly higher in more acidic areas of the tumour. This experiment reproduced our *in vitro* data *in vivo* by showing that LAMP2 expression was highest in acidic pH regions.

### Buffer therapy reduces LAMP2 expression

**Figure 5:**
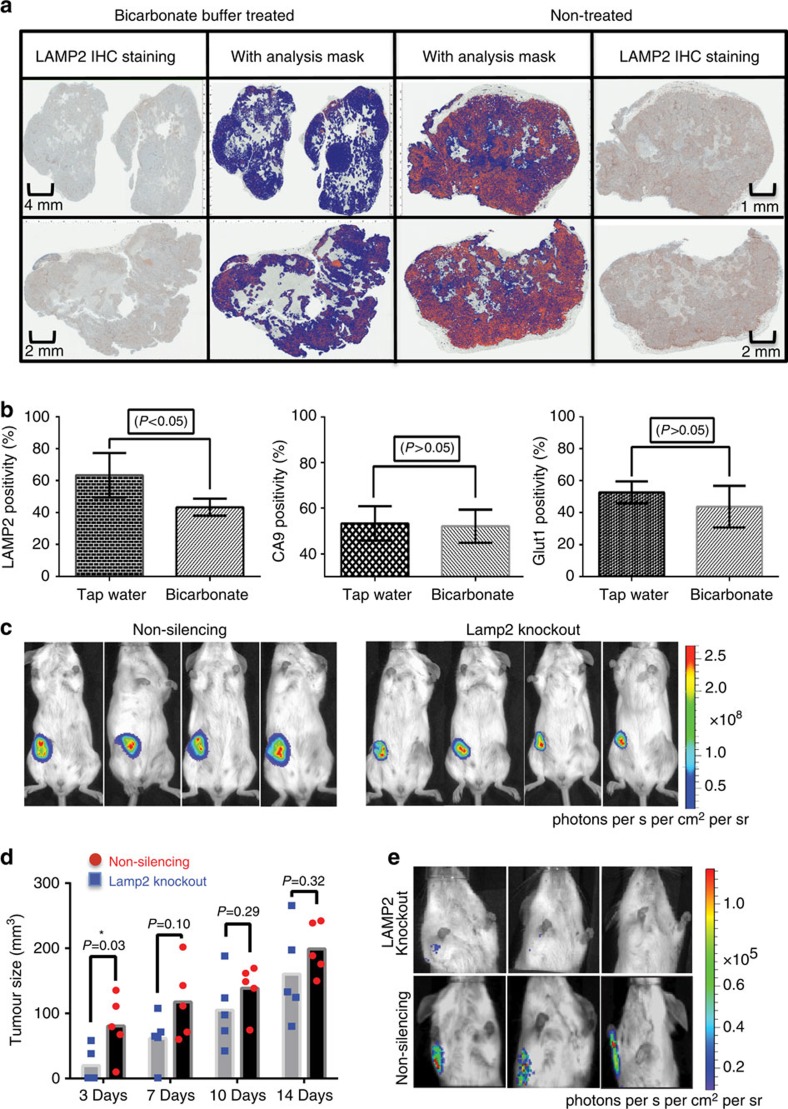
Effect of NaHCO_3_ buffer therapy on LAMP2 expression. (**a**) IHC staining of LAMP2 in representative cross-sections of ZR-75.1 tumours treated with tap water and NaHCO_3_ (left) or tap water (right), respectively. Positive pixel analysis of LAMP2 staining was carried out using Aperio Positive Pixel Count v9 (two columns in the middle; red is strong positive). (**b**) Positive pixel analysis of LAMP2 was compared with GLUT1 and CA9 as controls. A significant decrease in the percentage of strong positive LAMP2 pixels was observed in NaHCO3-treated cross-sections with much less decrease in GLUT1 expression and no significant change in CA9 expression. The data are plotted as the mean+s.d.) of six tumour per mouse from each group. (**c**) Bioluminescence images of luciferase activity in the primary tumours of MDA-mb-231/Luc with non-silencing shRNA (left) versus LAMP2 knockdown clone D5 (right) xenografted in nude mice. LAMP2 knockdown tumours had less activity and grew more slowly. (**d**) Tumour size comparison of knocked down LAMP2 clones versus the non-silencing group using caliper. LAMP2 knockdowns had very slow growth rate at the early stage of tumour growth. (**e**) Representative images of metastases into axillary lymph node of LAMP2 knockdown cells (*N*=5 mice) and non-silencing vector cells (*N*=5 mice). Knocking down LAMP2 reduces the metastasis of cancer cells significantly (Student's *t*-test, *P*<0.001, error bars represents mean with s.d.).

A key experiment to test whether LAMP2 is a marker of acidosis was performed by treating animals with oral buffers, for example, sodium bicarbonate (NaHCO_3_), which are known to neutralize tumour acidity. We have previously shown that oral administration of bicarbonate buffer alkalinizes the extracellular pH of xenografted tumours without affecting systemic pH^33^,^34^,^35^,^36^,^37^. In the current experiment, we chronically treated mice with orthotopic ZR-75.1 tumours with 200 mmol l^−1^ *ad lib* NaHCO_3_ for 2 weeks following tumour innoculation. As in our other studies, tap water was used for control animals. After harvesting the tumours and IHC staining, a positive pixel analysis was applied on both groups using trained software. The percent of LAMP2-positive pixels in ZR-75.1 bicarbonate-treated tumours was significantly (Student's *t*-test; *P*<0.05) less compared with the tumours in tap-water-treated animals (Fig. 5a,b). These data were consistent with our hypothesis that increased LAMP2 expression is upregulated by the acidic pH of tumours. To examine that the observed decrease of LAMP2 in bicarbonate-treated animals was not simply reduced epitope availability, we also stained for the HIF-1 clients GLUT1 and CA9, which were previously shown to be unaffected by buffer therapy^19^. As shown in Fig. 4, GLUT1 or CA9 levels were not significantly different between bicarbonate-treated animals and controls.

### Effect of LAMP2 knockdown on early tumour growth

Knowing that early tumour xenografts are acidic due to lack of vasculature leads us to test whether LAMP2 is critical for growth of early tumours. To study this, we created stable MCF-7 and MDA-MB-231 LAMP2 knockout cells using shRNA. Knockdown status was verified by western blot. In MDA-MB-231 cells, clones B5, B7, C7 and D5 showed the lowest expression among all the selected clones (Supplementary Fig. 5A). To find the best candidate for animal injection, we determined which of these clones was most sensitive to acid-induced cell death compared with non-shRNA-treated parental cells. As shown in Supplementary Fig. 5B, the MDA-MB-231 clone D5 showed the highest sensitivity to acidic media. We performed the same experiment on MCF-7, and clones C2 and D2 showed the most sensitivity to acidosis. However, because of their very low growth rate, we used MDA-MB-231 clone D5 for our *in vivo* studies. Five female mice were injected with LAMP2 knockdown MDA-MB-231/Luc cells (clone D5) as the experiment group and five female mice injected with non-silencing shRNA transfectants as controls. The size of the tumour was measured twice weekly using a caliper (Fig. 5c). We observed that the tumour size was smaller in the LAMP2 knockdown group in the initial phase of tumour growth (day 1–7). However, after day 7, there was no significant difference between two groups (Fig. 5d). There could be many reasons responsible for this, including selection for non-shRNA-expressing clones. We also investigated the activity and metastasis of these two groups using bioluminescence imaging of luciferase activity in the primary tumours (Fig. 5e). Our results showed that LAMP2 knockdown tumours were less active and smaller at the early stages, similar to the caliper data for tumour size. It also revealed that they have significantly fewer metastases in LAMP2 knockdown groups compared with controls.

### LAMP2 in breast cancers

**Figure 6:**
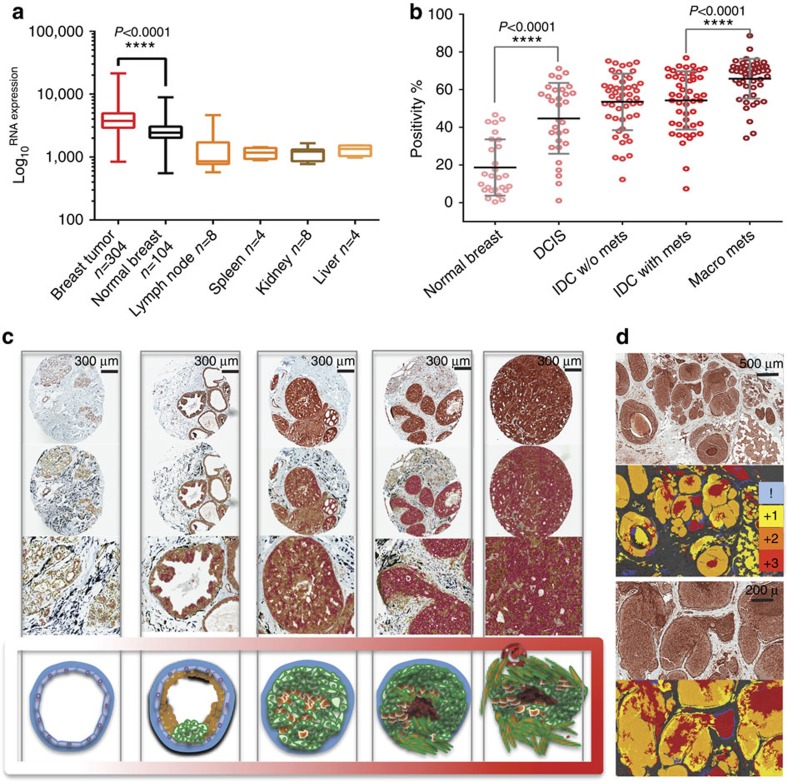
Translational study for LAMP2 expression in breast cancer samples. (**a**) Microarray mRNA expression profile of LAMP2 in breast cancer tumours and normal tissue samples. Data are represented as mean+s.d. Note the log10 scale of RNA expression. (**b**) Percent positivity of LAMP2 IHC staining of cores from the breast cancer patient samples TMA containing 201 biopsy cores. A consecutive raise in LAMP2-positive staining pattern was observed with the progress of breast tumours from DCIS to invasive ductal carcinoma with metastasis. (**c**) Representative images of analysed TMA cores from each stage with the corresponding illustration from the schematic model of breast cancer progression at the bottom. (**d**) Overexpression of LAMP2 in acidic regions of breast cancer tumours. IHC staining of a DCIS with LAMP2 antibody showed overexpression of this protein in regions expected to be more acidic such as centres of DCIS or DCIS with microinvasion. Underneath each IHC panel is a colour-coded picture of the IHC section based on LAMP2 positivity (0–3) for better understanding.

We then sought to investigate LAMP2 expression in breast cancer patient tumours. On the basis of our previous findings^1^,^5^, we hypothesized that an acidity biomarker should have two characteristics. First, as the rate of glycolysis increases with breast cancer progression, this should also lead to an association of progression with acidity^11^,^38^. Hence, a biomarker of acidosis should increase with stage. Second, this marker should exist in the regions of tumours that are far from vasculature or adjacent to necrosis and be co-localized with GLUT1, which contributes to production of acidosis^39^,^40^,^41^. Hence, we should observe an acidity marker to be peri-luminal in late-stage ductal CIS (DCIS). To test these, we first analysed gene expression microarray data from 304 breast cancers and adjacent normal tissues. The mRNA expression level of LAMP2 in breast tumours was significantly higher in cancers than in normal breast or other tissues (Fig. 6a, note log scale). Because mRNA levels are not necessarily related to the protein levels and localization, protein expression of LAMP2 was investigated by IHC of TMAs on patient sample biopsies from different stages of breast cancer. We stained 201 cores in a TMA for LAMP2. The expression of LAMP2 protein showed statistically (*P*<0.0001) higher levels in tumour samples, compared with normal breast. We then measured the positivity of each core for LAMP2 in different stages of breast cancer. Increased LAMP2 expression correlated with increased tumour progression from DCIS to invasive ductal carcinoma (Fig. 6b,c). There were notably significant (*P*<0.0001) differences between normal breast and DCIS, and between IDCs and metastases.

**Figure 7:**
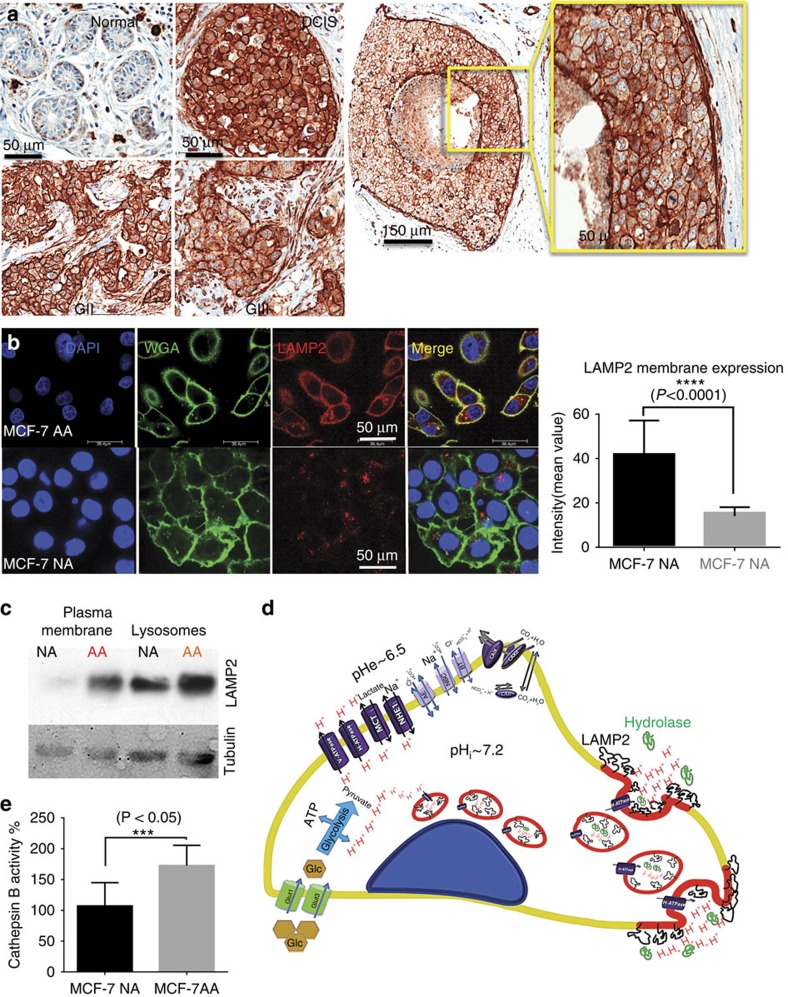
Proposed acid-adaptation mechanism. (**a**) LAMP2 expression in normal and cancer cells in breast tumour samples. LAMP2 membrane overexpression is indicated by arrows in breast cancer tumours (DCIS) in the right-most image that is a zoom-in of the yellow box in its neighbour. (**b**) LAMP2 expression in chronically acid-adapted cells versus non-adapted by ICC. To make sure LAMP2 is at the cell surface and not just cell periphery, we added wheat germ agglutinin (WGA) as a membrane marker to the cells; co-registration of LAMP2 and WGA was measured (merge) and confirmed membrane expression of LAMP2. (**c**) Membrane expression of LAMP2. Western blot of membrane protein extracted from AA and NA MCF-7 cells shows higher expression of LAMP2 at the cell surface of AA cells. (**d**) Schematic presentation of LAMP2 role in acid adaptation of cancer cells. In chronic acidosis, a possible mechanism could involve the exocytosis of more acidic lysosomes. This strategy helps the cells to secrete large amounts of acid in one step, and also by presenting of LAMP2 at the cell membrane protects themselves from acid degradation. LAMP2 with hydrated hyper-branched carbohydrate chains can protect the membrane against acidity. (**e**) The cathepsin-B assay in the media of AA and NA MCF-7 cells; AA MCF-7 cells secrete significantly more cathepsin B into their extracellular environment, consistent with increased lysosomal turnover rates.

To better investigate the biodistribution of LAMP2 protein, we stained whole-mount breast cancer samples from patients of different stages (I–IV). We consistently saw high LAMP2 staining in regions of tumours expected to be acidic, such as the peri-luminal region of DCIS and cells in areas of microinvasion (Figs 6d and 7b; Supplementary Figs 6A and 7A), which are consistent with our previous observation in DWC experiments that invasion is stimulated by acidosis^8^. To further investigate the localization of LAMP2, we again used GLUT1 as a positive control (Supplementary Fig. 6B). GLUT1 is present in glycolytic areas that are expected to be acidic. LAMP2 overexpression is present in highly glycolytic areas that were identified with GLUT1 staining in patient samples (colour-coded red in Fig. 6d and the right side in Supplementary Fig. 6A,B). We have shown previously that acidic regions are commonly observed at the invasive edges of tumours^8^. Staining whole-mount tumour samples for LAMP2 revealed that it is localized at the tumour–stromal interface and peri-luminal regions of DCIS, which are expected to be acidic (Supplementary Fig. 7A,B).

### Expression of LAMP2 at cell membrane

Interestingly, during the analysis of IHCs from patient samples and TMAs, we noted that LAMP2 was mostly associated with the PMs and not the lysosomes *per se*. In normal cells, LAMP2 is peri-nuclear and cytoplasmic, presumably associated with lysosomes and late endosomes (Fig. 7a). To further investigate this, we examined the membrane expression of LAMP2 in acid-adapted and acid-naive cells at high resolution using ICC (Fig. 7b; Supplementary Fig. 8). These results showed higher expression of LAMP2 at the cell membrane of AA MCF-7 cells compared with NA. To further confirm the PM expression of LAMP2, we isolated the membrane proteins of AA and NA MCF-7 cells followed by a western blotting (Fig. 7c) and these also showed elevated PM expression of LAMP2. Considering that the main role of LAMP2 as a glycoprotein is to protect the lysosome membrane against intra-lysosomal acidity^42^ and that we have observed localization of this protein at the cell membrane, we propose that this represents a new defence mechanism for acid adaptation in cancer cells: that the localization and overexpression of LAMP2 at the cell membrane makes acid-adapted cells resistant to damage by extracellular acidosis and probably acid-activated lysosomal hydrolases released into the ECM. With the lysosomal membranes on the surface, one might expect increased release of lysosomal enzymes into the medium in AA cells (Fig. 7d). To test this, we assayed cathepsin B (a lysosomal enzyme) in the media of AA and NA MCF-7 cells. AA MCF-7 cells secrete significantly more cathepsin B into their extracellular environment (Fig. 7e). If LAMP2 membrane expression is a result of lysosomal turnover, we might be able to interfere with this process using Chloroquine. Therefore, NA and AA MCF-7 cells were treated with 50 μM Chloroquine. ICC of Chloroquine-treated cells with LAMP2 antibody showed that Chloroquine did lower the membrane expression of LAMP2 in acid-adapted cells (Supplementary Fig. 9).

### Effect of acid adaptation on low-pH survival and competition

We propose that cell surface expression of LAMP2 provides a novel protection mechanism for cells exposed to low-pH conditions. To test this, we treated MCF-7, ZR-75.1 and MDA-mb-231 cells that were either AA or NA (which, respectively, do and do not express plasmalemmal LAMP2) to highly acidic (pH 5.0) conditions for 96 h. As shown in Supplementary Fig. 10, there was significantly higher survival in the acid-adapted MCF-7 and ZR-75.1 cells compared with controls. In contrast, acid adaptation had little effect on survival of MDA-MB-231. Notably, these cells appear to express LAMP2 at their PM even under control conditions (Supplementary Fig. 10B).

Next, we studied whether acid adaptation gives any advantage to the cells in populations. To investigate this, we transfected NA MCF-7 cells with green fluorescent protein (GFP)-expressing plasmids and AA MCF-7 with red fluorescent protein and co-cultured them as hanging-drop 3D spheroids. We consistently observed that AA cells (red) were preferentially at the edge, and NA cells (green) were at the centre of spheroids and then, with time, the AA outgrew the NA cells and took over the majority of the population within the spheroid (Supplementary Fig. 11). This is compatible with what we observed previously in window chamber experiment: that is, that AA cells moved to the edge. We speculate that this behaviour imparts a defensive mechanism at the leading edges of the tumours.

## Discussion

Altered metabolism is one of the hallmarks of cancer^43^. As a consequence, acidosis is commonly observed in solid tumours. It is a first principle of evolution that commonly observed phenotypes must confer a selective advantage; but in the case of tumour acidity, this can only occur if the cancer cells become adapted to low-pH growth. Tumour development occurs through interactions between cancer cells and their microenvironment, and compelling evidence indicates that the tumour–stromal interface is acidic. Acidosis forces cancer cells to reprogramme to survive in this environment, which subsequently imparts on them an evolutionary advantage over acid-naive stromal cells. In spite of the importance of acidosis in cancer biology and clinical studies, a well-accepted marker for acidosis and acid adaptation is lacking. Using SILAC proteomics for discovery, we identified a number of candidate proteins that are upregulated in acid-adapted cells. One of these, LAMP2 was characterized as behaving in a manner consistent with acid adaptation and hence, may have practical use to design new imaging, diagnostic or theragnostic markers for the acidic niche and cells adapted to this milieu. Such a marker could be used immunohistochemically to quantify the presence of acidity in biopsies, or may be targeted with magnetic resonance imaging or positron emission tomography molecular imaging probes for *in vivo* diagnostics. Such agents also have the potential to serve broadly for the non-invasive detection of acidic regions of tumours, which are associated with invasion/metastasis as well as resistance to therapy. They may also predict response to acid-modulated therapies (for example, acid-sensitive caged compounds).

The discovery of LAMP2 as a biomarker and further observation of its presence on cell surface of acid-adapted cells has also led us to propose a new adaptation mechanism for chronic acidosis in solid tumours, such as breast cancer. It is known that solid tumours are acidic and cancer cells have an advantage of growing in the acidic environment over normal and stromal cells. It has been previously shown that lysosomal turnover increases in response to acidosis, and there are acid-activated lysosomal proteases in the ECM that remodel ECM components such as collagen, which also play a role in cancer cell invasion^11^. It is not known, however, how cancer cells themselves could survive in this condition that is poisonous for normal cells. Herewith we are proposing a new mechanism wherein cancer cells evolutionary adapt to chronic acidosis: increasing the turnover of lysosomes and exosomes helps the cells pump the extra protons of cytoplasm into the lysosomes using ATPase proton pumps and stabilize the cytoplasmic pH, pHi. Exocytosis of these lysosomes release acid and hydrolases into the ECM, while also retaining some of their components such as LAMP2 incorporated in the PM. We propose that expression of LAMP2 on the PM increases survivability in the acidic environment. As an important component of acid adaptation, LAMP2 expression at cell surface of acid-induced cancer cell can provide a novel therapeutic target. However, we still do not know the molecular mechanism of this process and that is our future direction of this study.

## Methods

### Cell culture and acid adaptation *in vitro*

MCF-7, MCF10-DCIS, MDA-MB-231 and ZR-75.1 cells were acquired from American Type Culture Collection (ATCC, Manassas, VA, 2007–2010) and were maintained in DMEM-F12 (Life Technologies) supplemented with 10% fetal bovine serum (HyClone Laboratories). Growth medium was further supplemented with 25 mmol l^−1^ each of PIPES and HEPES and the pH adjusted to 7.4 or 6.7. Acid-adapted cells were monitored by microscopy and confirmed to maintain their original morphology. Cells were tested for mycoplasma contamination and authenticated using short tandem repeat DNA typing according to ATCC's. To achieve acid adaptation, cells were chronically cultured and passaged directly in pH 6.7 medium for ∼3 months. Chronic low-pH-adapted cells underwent about 30 passages. Acid-adapted cells had similar growth rates to non-adapted cells.

### SILAC labelling

Acid-adapted and naive cells were labelled by SILAC. Cells were cultured in heavy SILAC media (Δ6-lysine and Δ10-arginine) for eight doublings. Extent of labelling was determined by LC–MS/MS analysis of tryptic peptides from labelled samples to ensure >98% labelling.

### Lysis and digestion

Cells were lysed by sonication in a buffer of 50% trifluoroethanol and 50 mM ammonium bicarbonate, pH 8.0, and protein was measured by the Bradford method. Protein from heavy- and light-labelled cells was combined in equal amounts, and lysis buffer was added to bring the final volume to 200 μl. The combined protein was reduced with 100 μl of 40 mM TCEP/100 mM dithiothreitol for 1 h at 37 °C. Proteins were alkylated with 100 μl of 200 mM iodoacetamide for 30 min in the dark at ambient temperature. The volume of the reduced and alkylated sample was brought to 1 ml with 50 mM ammonium bicarbonate, pH 8.0. Trypsin was added at a ratio of 1:50 and samples were digested at 37 °C overnight. Digests were frozen at −80 °C and lyophilized. Dried peptides were resuspended in HPLC water with 0.1% TFA and desalted on 100-mg Thermo hypersep C18 columns. Eluted peptides were dried in a Speed-Vac and resuspended in HPLC water for isoelectric focusing fractionation.

### Isoelectric focusing fractionation

Tryptic peptides were fractionated using a narrow-pH-range fractionation strategy previously described^44^. At the end of the isoelectric focusing programme, strips were manually cut into 20 fractions. Peptides were extracted and samples were combined in the following manner to achieve 15 fractions for LC–MS/MS analysis: (anode end) samples 1–2, 3–4, 5–6, 7–8 and 9–10 were combined to make five fractions, samples 11–20 were left as individual fractions.

### LC–MS/MS

Samples were analysed as duplicate injections for each fraction. A nano-flow ultra-high performance liquid chromatograph (RSLC, Dionex, Sunnyvale, CA) coupled to an electrospray ion trap mass spectrometer (LTQ-Orbitrap, Thermo Scientific, San Jose, CA) was used for tandem MS peptide-sequencing experiments. The sample was first loaded onto a pre-column (2 cm × 75 μm ID packed with C18 reversed-phase resin, 5 μm particle size, 100 Å pore size) and washed for 8 min with aqueous 2% acetonitrile and 0.04% trifluoroacetic acid. The trapped peptides were eluted onto the analytical column, (C18 Pepmap 100, 75 μm × 50 cm ID, Dionex). The 120-min gradient was programmed as: 95% solvent A (2% acetonitrile+0.1% formic acid) for 8 min, solvent B (90% acetonitrile+0.1% formic acid) from 5 to 15% in 5 min, 15 to 40% in 85 min, then solvent B from 50 to 90% B in 7 min and held at 90% for 5 min, followed by solvent B from 90 to 5% in 1 min and re-equilibration for 10 min. The flow rate on the analytical column was 300 nl min^−1^. Ten tandem mass spectra were collected in a data-dependent manner following each survey scan. The MS scans were performed in the Orbitrap to obtain accurate peptide mass measurements, and the MS/MS scans were performed in the linear ion trap using a 60-s exclusion for previously sampled peptide peaks. Mascot (www.matrixscience.com) searches were performed against the UniProt human database downloaded on 11 July 11 2012. Two missed tryptic cleavages were allowed, the precursor mass tolerance was 1.2 Da to accommodate selection of different isotopes of the peptide precursor. MS/MS mass tolerance was 0.6 Da. Dynamic modifications included carbamidomethylation (Cys), oxidation (Met), heavy lysine (Δ6) and heavy arginine (Δ10).

Quantification of differences in protein expression between SILAC-labelled samples was performed as described using MaxQuant^25^,^45^. Results were filtered to require a posterior error probability (PEP) score <0.05 and summed intensity >0. Candidates were selected among proteins that consistently showed at least a 1.5-fold increase under low-pH conditions across label-flipping experiments.

### LC-MRM

Using a nanoLC system (Easy-nLC, Thermo Scientific), 200 ng of unfractionated tryptic digest was loaded onto a trap column at 5 μl min^−1^ (C18PepMap100, Dionex, 300 μm × 5 mm), washed for 20 min, then eluted onto a column (75 μm × 15 cm ID, 3 μm particle size and 100 Å pore size Dionex). Peptides were eluted over a 25-min gradient (from 5–50% B) at a flow rate of 300 nl min^−1^. The solvent system was composed of aqueous 2% acetonitrile with 0.1% formic acid (A) and aqueous 90% acetonitrile with 0.1% formic acid (B). LC-MRM was performed on a TSQ Quantum Ultra triple quadrupole mass spectrometer (Thermo Scientific) spraying from 10-μm tips (New Objective, Woburn, MA) at 2,500 V with 200 °C transfer tube temperature, and 12 V skimmer offset. Acquisition parameters were as follows: Q1 was *m*/*z* 0.4 in width, Q3 was *m*/*z* 0.7 in width and each transition was monitored for 20 ms. Fragmentation was achieved with 1.5 mTorr argon. A minimum of three transitions was measured for each peptide. Quantification was achieved using the sum of the peak areas for all detected transitions using Skyline^27^. The ratio of peak area of the endogenous peptide divided by the corresponding internal standard was calculated for each peptide in the sample to enable quantification of protein expression levels.

### Transfections

For transfection of siRNA, cells were seeded in the cultures (5,000 cells per well in 96 wells) 3 days before the transfection. LAMP2 siRNAs (Trilencer-27: 27mer siRNA, Origene) were transfected using Lipofectamine RNAiMAX (Invitrogen # 13778030) according to the manufacturer's protocol. To establish stable cell lines, the MCF-7 and MDA-MB-231 cells were infected with Plasmids expressing shRNA to LAMP2 (TR311794, Origene), LAMP2 overexpressing (RC221216, Origene) and red fluorescent protein or GFP expressing using Fugene 6 (Promega) at an early passage and were selected using 2 μg ml^−1^ puromycin (Sigma).

### Western blotting

Acid-adapted and non-adapted DCIS, MCF-7, ZR-75.1 and MDA-MB-231 cells were grown with the same number of passages and used for whole-protein extraction. Lysates were collected using Insect Cell Lysis Buffer (554778; BD Biosciences) and RIPA buffer containing 1 × protease inhibitor cocktail (P8340; Sigma-Aldrich). Twenty micrograms of protein per sample was loaded on polyacrylamide–SDS gels, which later were electrophoretically transferred to nitrocellulose. Membranes were incubated with primary antibodies against rabbit polyclonal LAMP2 (1:1,000, ab37024 Abcam), rabbit poly clonal S100A6 (1:1,000, ab 75676 Abcam) and GAPDH (1:4,000, antirabbit; Santa Cruz Biotechnology). Full blots are shown in Supplementary Fig. 12.

### Immunofluorescence

Cells cultured at pH 6.7 chronically and pH 7.4 of with the same passage were rinsed with PBS, fixed in cold 4% paraformaldehyde for half an hour and then blocked with 4% bovine serum albumin in PBS. Samples were incubated with LAMP2 rabbit polyclonal primary antibody (1:100; ab 37024 Abcam) and secondary Alexa-Fluor 488 antirabbit (1:500) antibody. Coverslips were mounted using ProLong Gold Antifade Reagent (Life Technologies) and images were captured with a Leica TCS SP5 (Leica) confocal microscope.

### Dorsal window chamber

Tumour constructs were prepared using the tumour droplet method^34^. MCF-7, ZR-75i, MCF-7 (GFP) or MDA-MB-231 (GFP) cells were suspended in 0.8 mg ml^−1^ of type-1 collagen (BD Bioscience #354249) and 1 × DMEM at a final concentration of 2.5 × 10^6^ cells ml^−1^. Then, a 48-well non-tissue culture plate was used to place 5–15-μl drops (depends on the size of the tumour) of the tumour suspension in it. The droplets were polymerized in the centre of the well after 20–30 min of incubation at 37 °C. Next, 25 μl of 1.25 mg ml^−1^ collagen 1 was added to the surrounding of the tumour droplets. This helped the tumour droplets to have nicely defined borders. Then 200 μl of growth medium (DMEM/F12 with 10% fetal bovine serum) was added to the wells and incubated in 37 °C.

Meanwhile, a DWC was prepared to implant into the recipient mice for nest 24–48 h of culturing the tumour droplet constructs. Constructs were added aseptically into the wound area right after the surgery. Intravital microscopy was started from 24–48 h after surgery to acquire tumour integrity and growth. To develop a pH map for each tumour and its microenvironment inside the chamber, we tail-vain-injected SNARF (a fluorescent molecule that has different emission in basic and acidic pHs) to the mice and imaged after 45 min. For SNARF imaging, we used excitation with an Argon laser tuned to 543 nm and emission was collected in 590–640 nm bandpass filter. Images were collected using an Olympus FV1000 MPE (multiphoton) microscope. Analysis was carried on using Image-Pro Plus v6.2 (Media Cybernetics; Bethesda, MD).

### Tumour development and buffer therapy in mouse model

All animals were maintained in accordance with IACUC standards of care in pathogen-free rooms, in the Moffitt Cancer Center and Research Institute (Tampa, FL) Vivarium. One week before inoculation with tumour cells, ZR-75.1 cells (1 × 10^7^) in the mammary fat pads, female nu/nu mice 6–8 weeks old (Charles River Laboratories) were placed in two cohorts per experiment, which were allowed to drink either tap water or 200 mM NaHCO_3_. ZR-75.1 tumours reached a tumour volume of 500 and 700 mm^3^, before the treatment with either tap water or 200 mmol l^−1^ NaHCO_3_. The weights of the water bottles were recorded before and after providing them to the animals, thereby tracking the amount of liquid consumed per cage. Animal weights were measured and recorded twice weekly, and the overall health of each animal was noted to ensure timely end points within the experiment. Finally, animals were humanely killed and tumours were extracted, fixed in 10% formalin, paraffin embedded and further processed for IHC.

### Microarray analysis

Affymetrix expression data for LAMP2 and S100A6 genes in patient samples were produced from publicly available data sets. The CEL files for the tumour samples were downloaded from the Gene Expression Omnibus (GEO) database (http://www.ncbi.nlm.nih.gov/geo/), data series GSE2109. Normal tissue data were from the GEO data series GSE7307, Human Body Index. The CEL files were processed and analysed using the MAS 5.0 algorithm (Affymetrix) and screened through a rigorous quality control panel to remove samples with a low percentage of probe sets called present by the MAS 5 algorithm, indicating problems with the amplification process or poor sample quality; high scaling factors, indicating poor transcript abundance during hybridization; and poor 30/50 ratios, indicating RNA degradation either before or during processing. The remaining samples were normalized to the trimmed average of 500 in the MAS 5 algorithm before comparison of the expression values across tumours and normal samples^46^.

### Immunohistochemistry

For mouse xenografts, tumours in the window chamber experiment were fixed in 10% neutral buffered formalin (Thermo Scientific) for 24 h, processed and embedded in paraffin. Routine haematoxylin and eosin stains were conducted on 4-μm sections of tissue. S100A6 and LAMP2 proteins were detected by IHC of their related antibodies using the Ventana Discovery XT automated system (Ventana Medical Systems). Rabbit polyclonal LAMP2 antibody (#ab37024 Abcam) and rabbit S100A6 antibody (Sigma HPA007575) were incubated at a dilution of 1:400. All antibodies were incubated for 32 min in Dako antibody diluent, followed by a 20-min incubation in Ventana OmniMap antirabbit secondary antibody and detected with the Ventana ChromoMap Kit system.

For human tissues, a TMA containing formalin-fixed and paraffin-embedded human breast tissue specimens was constructed in Moffitt Cancer Center histology core. The TMA contains 27 normal breast tissue, 30 DCIS, 48 invasive ductal carcinomas without metastasis, 49 invasive ductal carcinomas with metastasis and 48 lymph node macrometastases of breast cancer. Cores were selected from viable tumour regions and did not contain necrosis. A 1:400 dilution of anti-LAMP2 (#ab37024, Abcam), anti-GLUT1 and anti-S100A6 antibody (Prestige Antibodies Powered by Atlas Antibodies, Sigma-Aldrich) were used as primary antibodies. Positive and negative controls were used. Normal placenta was used as a positive control for LAMP2, normal breast was used as a positive control for GLUT1 and normal kidney was used as a positive control for S100A6. For the negative control, an adjacent section of the same tissue was stained without application of primary antibody, and any stain pattern observed was considered as nonspecific binding of the secondary.

Immunohistochemical analysis was conducted using digitally scanning slides and scoring by three independent reviewers. The scoring method used by the pathologist reviewer to determine (1) the degree of positivity scored the positivity of each sample ranged from 0 to 3 and were derived from the product of staining intensity (0–3^+^). A zero score was considered negative, score 1 was weak positive, score 2 was moderate positive and score 3 was strong positive. (2) The percentage of positive tumours stained (on a scale of 0−3).

### Statistical analysis

A two-tailed unpaired Student's *t*-test was performed to compare treated and non-treated groups of animals and also for tumours against normal cells to determine statistical significance. The significance level was set as *P*<0.05.

### Ethics statement

All procedures on animals were carried out in compliance with the Guide for the Care and Use of Laboratory Animal Resources (1996), National Research Council, and approved by the Institutional Animal Care and Use Committee, University of South Florida (IACUC# R4051).

Access to patient tissues samples was obtained following informed written consent of patients under a human subjects protocol (MCC16511) approved by the University of South Florida Institutional Review Board.

## Additional information

**How to cite this article:** Damaghi, M. *et al*. Chronic acidosis in the tumour microenvironment selects for overexpression of LAMP2 in the plasma membrane. *Nat. Commun*. 6:8752 doi: 10.1038/ncomms9752 (2015).

## Supplementary Material

Supplementary Information Supplementary Figures 1-12 and Supplementary Table 1

## Figures and Tables

**Figure 1:**
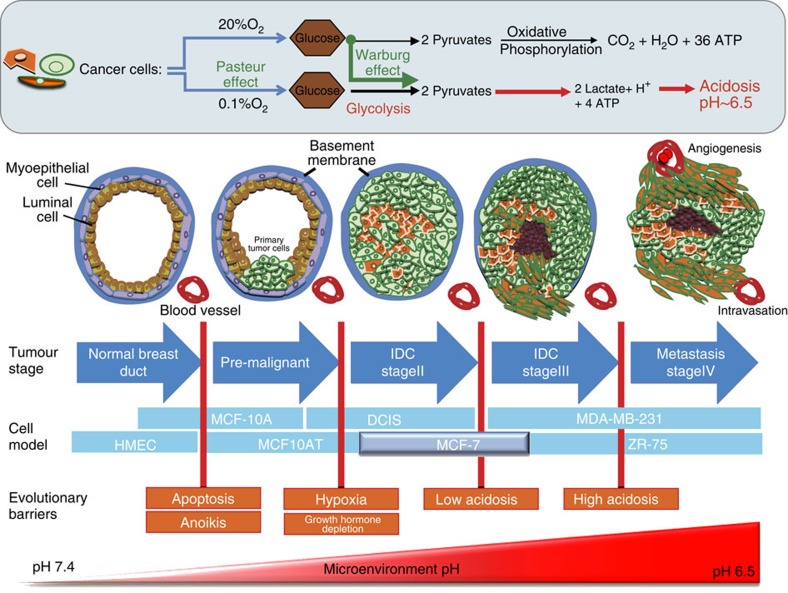
Effect of acidosis in tumour progression and development. Hypoxia, poor vasculature and increased flux of carbons through fermentative glycolysis leads to extracellular acidosis in solid tumours (Pasteur effect). Cancer cells can maintain the glycolytic phenotype even in the presence of oxygen (Warburg effect) causing further and constant acidification of the tumour microenvironment. The acidification and adaptation to acidosis starts at the centre of the duct, far from vasculature. Adaptation and development of resistance to intraductal acidosis is one of the key issues in cancer development and evolution that leads to a more aggressive phenotype. Evolutionary advantages over non-resistant cells, specifically normal epithelial and stromal cells are gained, such as being more migratory and glycolytic. This figure illustrates how acidosis increases during breast cancer progression from DCIS to Stage IV metastasis and how acid adapted cells (organge cells) become more aggressive and invasive.
