# Supplementary material for: Chronic acidosis in the tumour microenvironment selects for overexpression of LAMP2 in the plasma membrane
Source: Nat Commun. 2015 Dec 10;6:8752. doi: 10.1038/ncomms9752 (PMC4682176; doi:10.1038/ncomms9752)
Supplement: Supplementary Information — Supplementary Figures 1-12 and Supplementary Table 1 [file ncomms9752-s1.pdf]

Supplementary materials

Supplementary Figure 1.

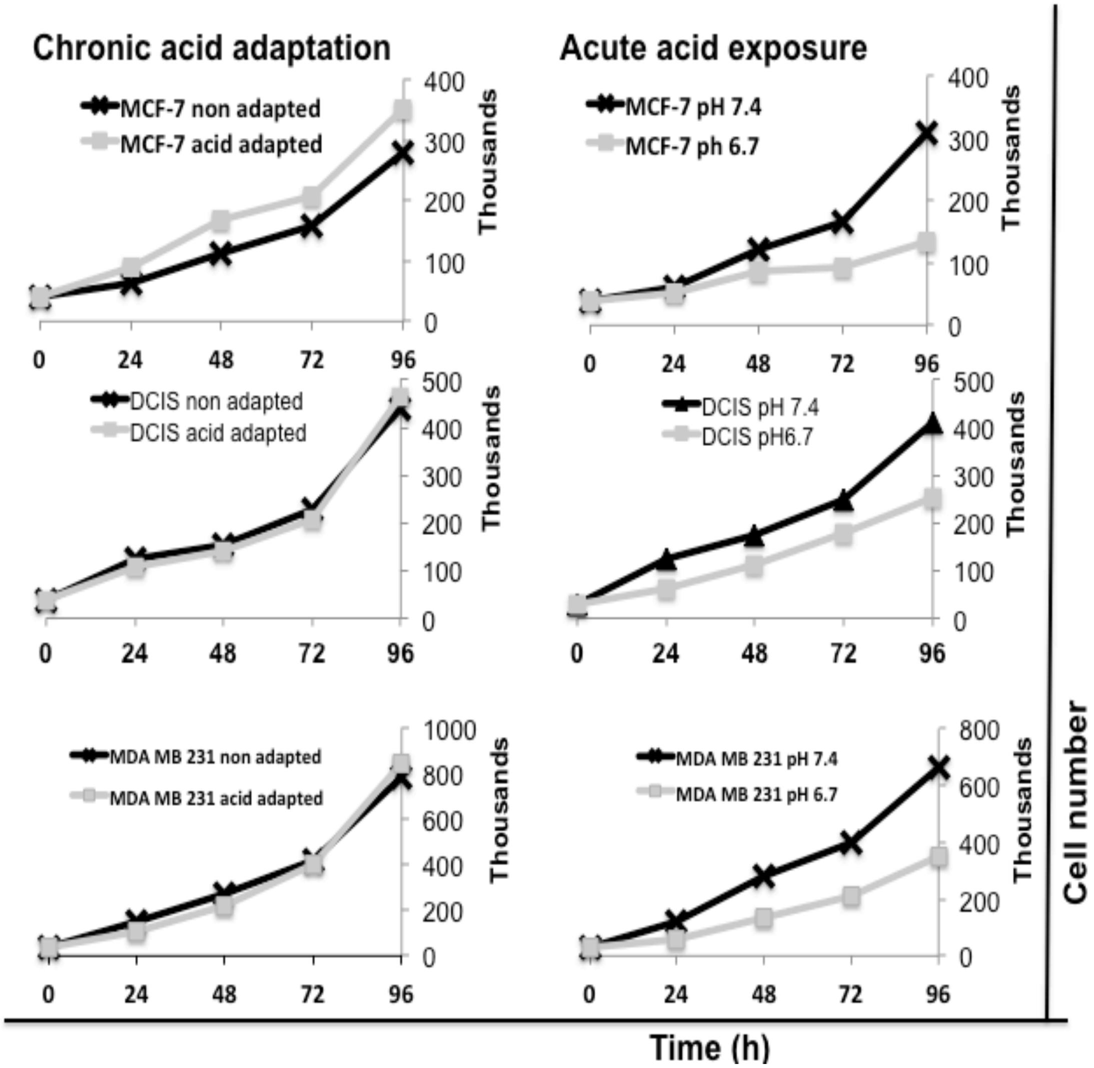

Proliferation assay of breast cancer cell lines. Left, proliferation assay of chronically acid adapted breast cancer cells vs. non-adapted ones, and right, proliferation assay for acid exposed cells (pH 6.7) vs. normal pH (pH 7.4) cells.

Supplementary Figure 2.

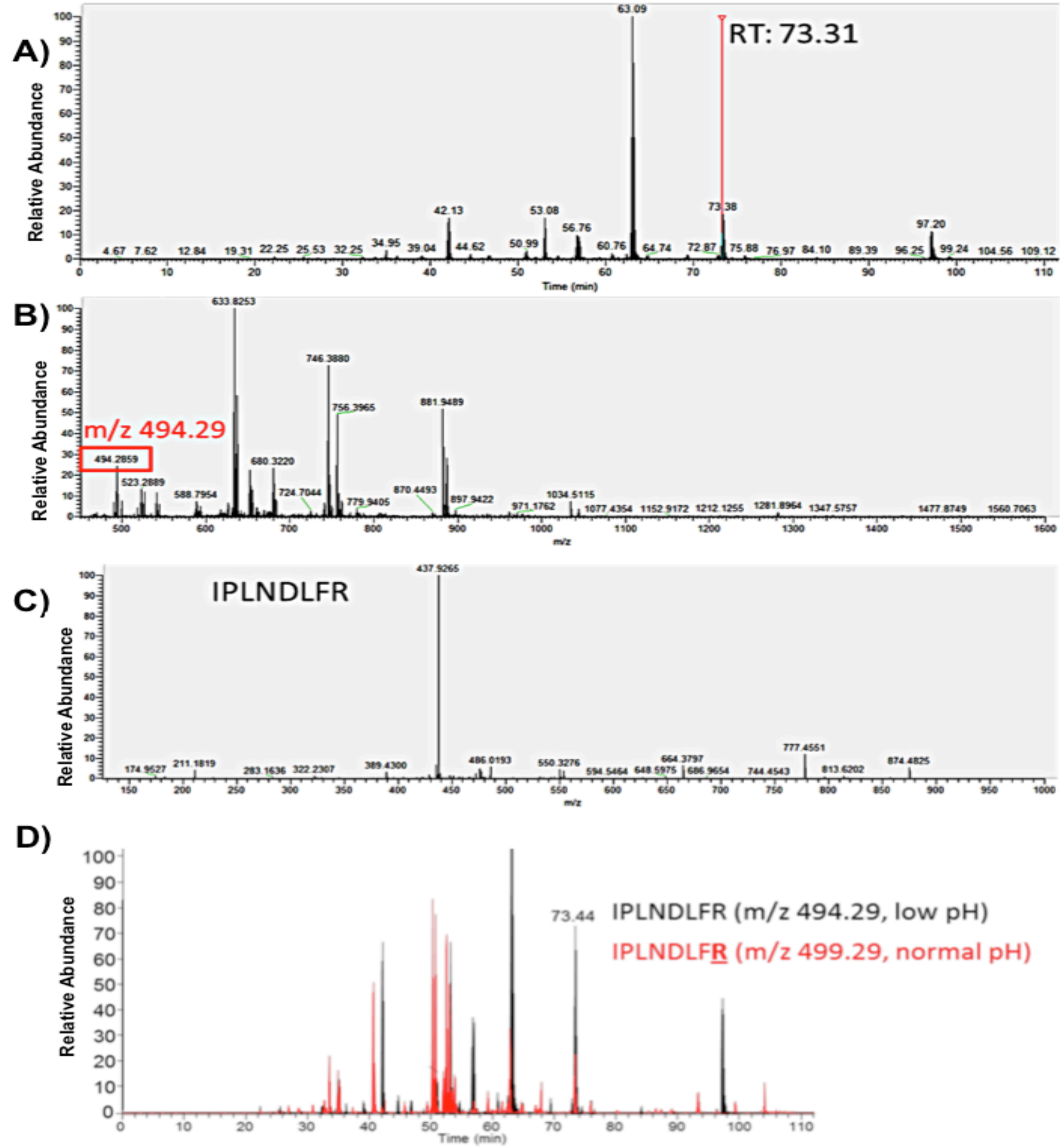

Shotgun proteomic analysis for identification of low pH-induced proteins. A) Chromatogram depicting base peak ion intensities across a 2-hour HPLC gradient. **B)** MS1 showing ions co-eluting at a retention time of 23.02 minutes. **C)** MS/MS fragmentation of the precursor ion at m/z of 494.29, which was identified as a doubly charged peptide, IPLNDLFR, from LAMP2. **D)** Extracted MS1 ion chromatogram is shown for peptide IPLNDLFR. Candidate low pH-induced proteins were identified by comparing the MS1 signal intensities for co-eluting light and heavy SILAC labeled peptide peaks using MaxQuant software. Bold underline in sequence indicates heavy isotope labeled residue

Supplementary Figure 3.

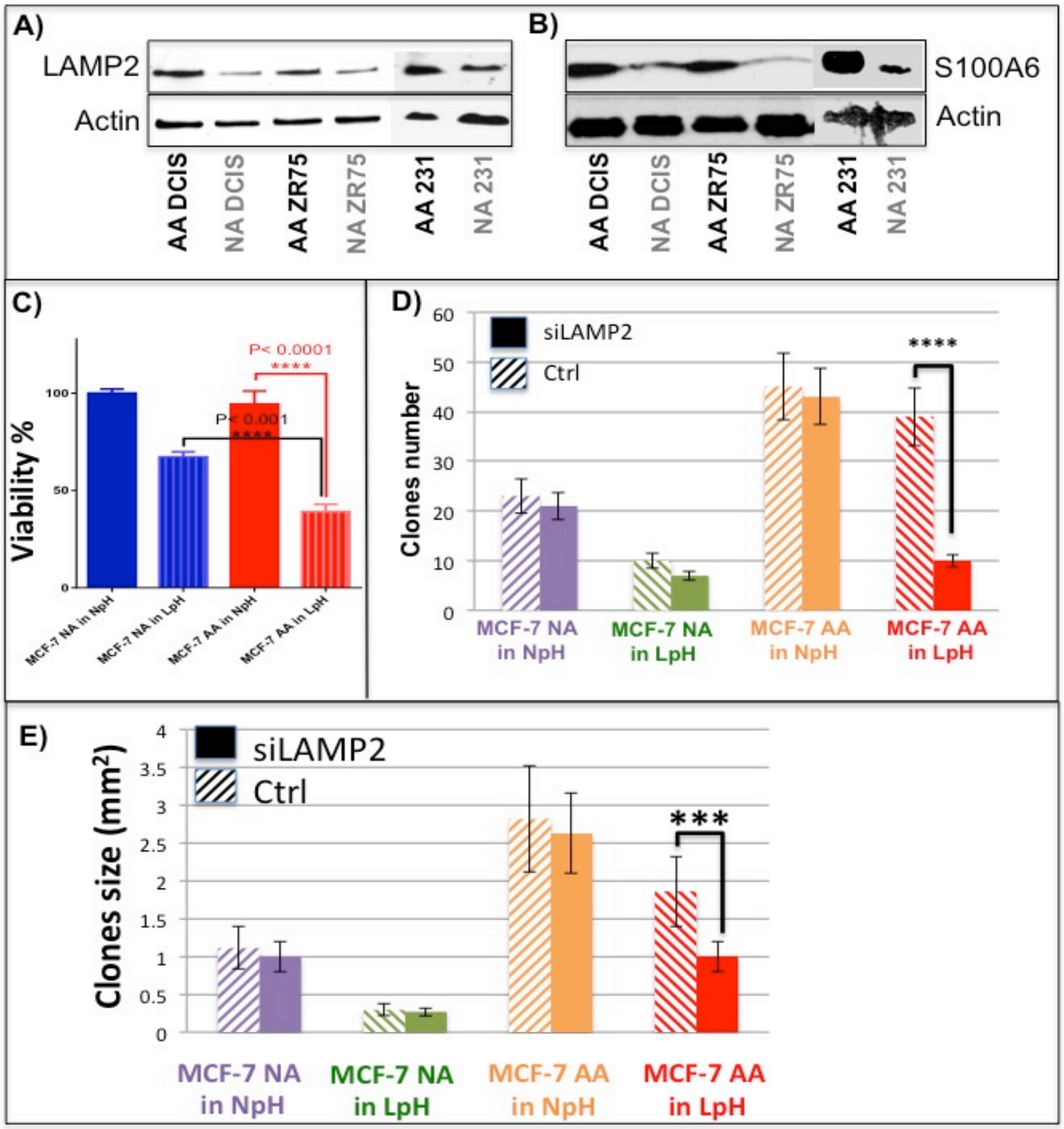

LAMP2 plays role in acid exposed and adapted cancer cells. A) Western blot of LAMP2 on AA- DCIS, ZR-75.1, and MDA-MB-231 shows higher expression of LAMP2 on acid adapted cells. **B)** S100A6 over expression in acid adapted cells is confirmed by western blotting in breast cancer cell lines. **C)** LAMP2 siRNA treatment of AA and NA MCF-7 cells in normal and low pH revealed that acid adapted cells are more related to LAMP2 and late knock down of LAMP2 is more effective on killing the acid adapted cells (data are analyzed using t test and $p<0.0001$). **D)** Average number of clones generated from 500 cells reveals fewer number of clones in LAMP2 siRNAs treated cells as compared to the control siRNA in MCF-7 cell line. **E)** Average size of clones generated from 500 cells reveals smaller clones size in LAMP2 siRNAs treated cells as compared to the control siRNA in MCF-7 cell line. For all the above graphs, data are represented as mean + Standard Deviation (SD).

Supplementary Figure 4.

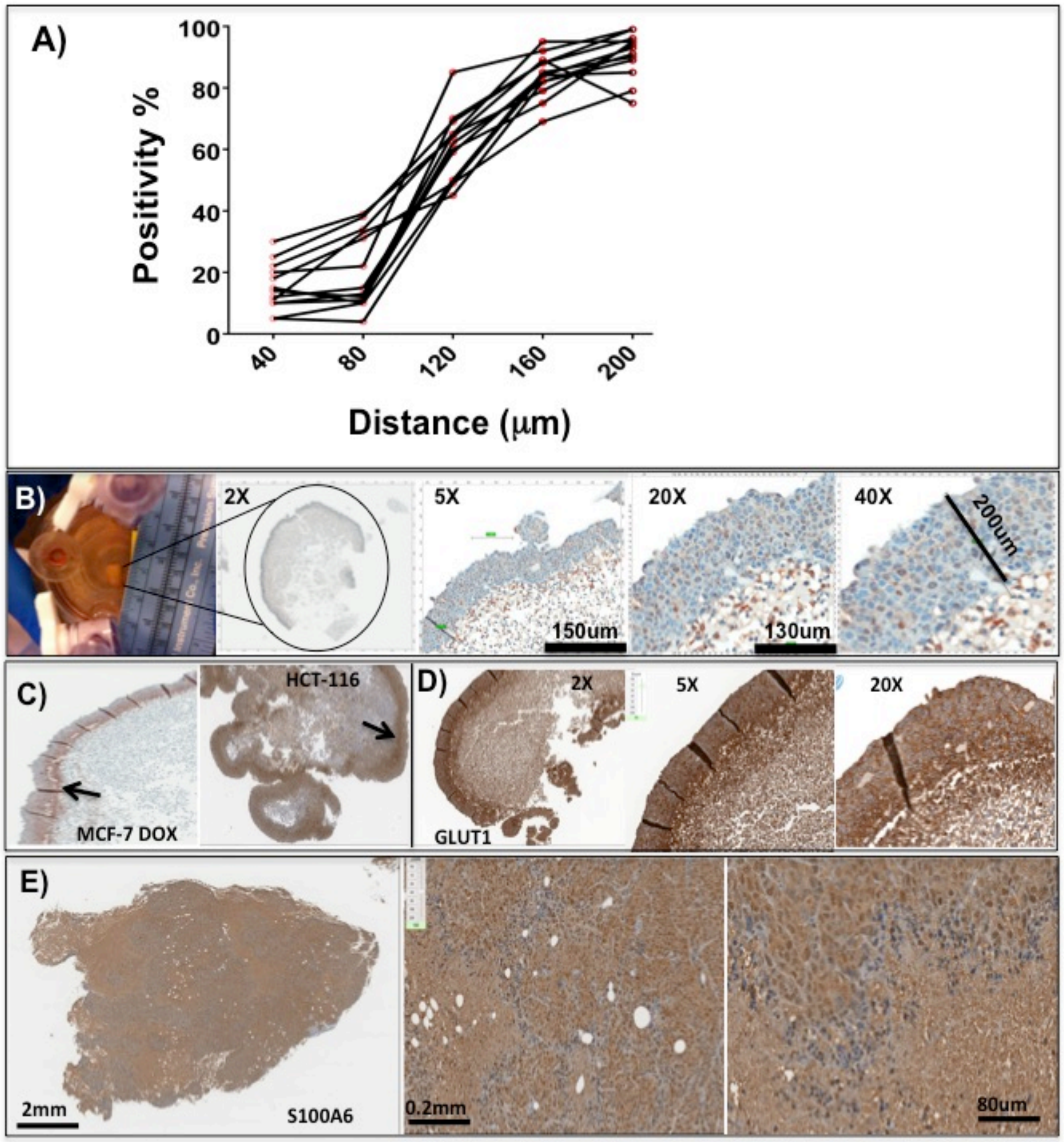

***In vitro* 3D and *In vivo* Validation of LAMP2 and S100A6. A)** Analysis of LAMP2 expression in MCF-7 cells spheroid 3D culture model (Rotary system). Increased expression of LAMP2 close to the central part of the tumors that has the highest acidity is observed. Each red dot is a spheroid used for analysis that different regions of which is connected through solid line. **B**) 3D culture of breast cancer cells as spheroid did not confirm the expression of S100A6 at the acidic regions. **C**) Elevated LAMP2 expression at the oxygen diffusion limit is also observed in spheroids made of other cancer cell lines such as MCF-7-DOX and HCT-116. **D**) Glut1 is used to indicate regions of increased glycolysis. GLUT1 expression at the acidic region of spheroids is elevated. **E**) Staining of xenografted tumors in mice with S100A6 antibody did not confirm the expression of this protein at acidic regions of the tumors.

Supplementary Figure 5.

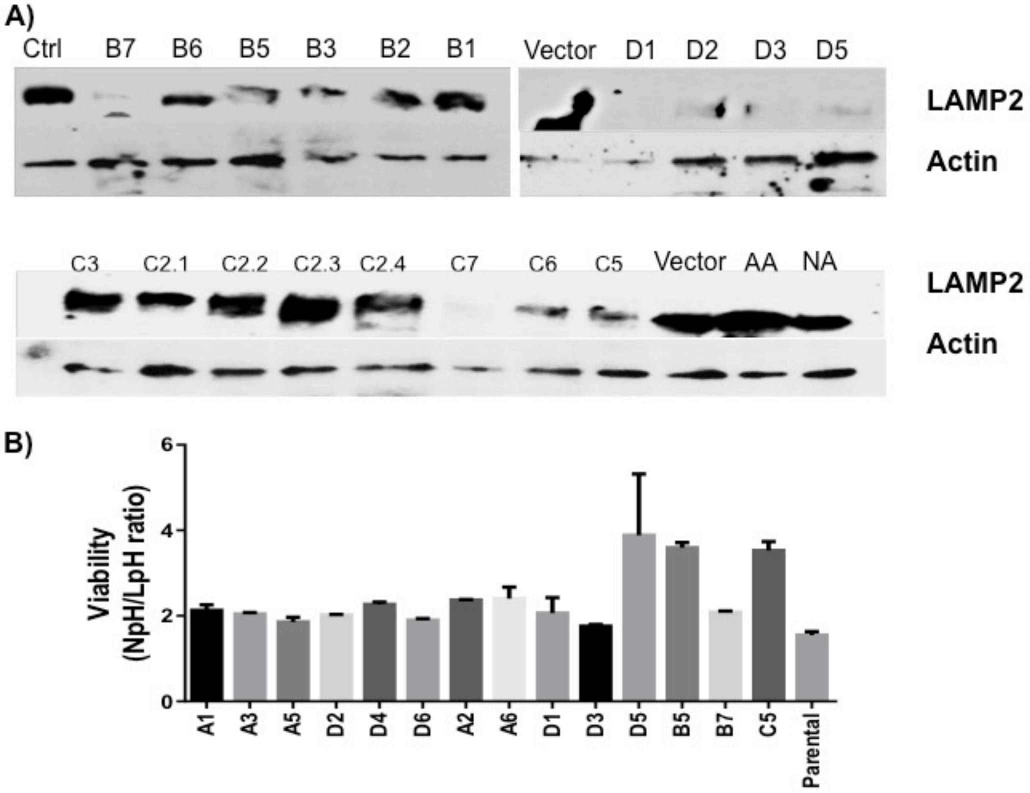

Knocking down LAMP2 decreases the acid tolerance of cancer cells. A) Western blot on shRNA LAMP2 clones. The clones with lowest LAMP2 expression were selected for acid resistance test. **B)** Acid killing test on LAMP2 Knocked out clones reveled the most sensitive clones to acidosis that later was injected to animals (data are triplicate and analyzed by t test and P=0.09 for Clone D5).

Supplementary Figure 6.

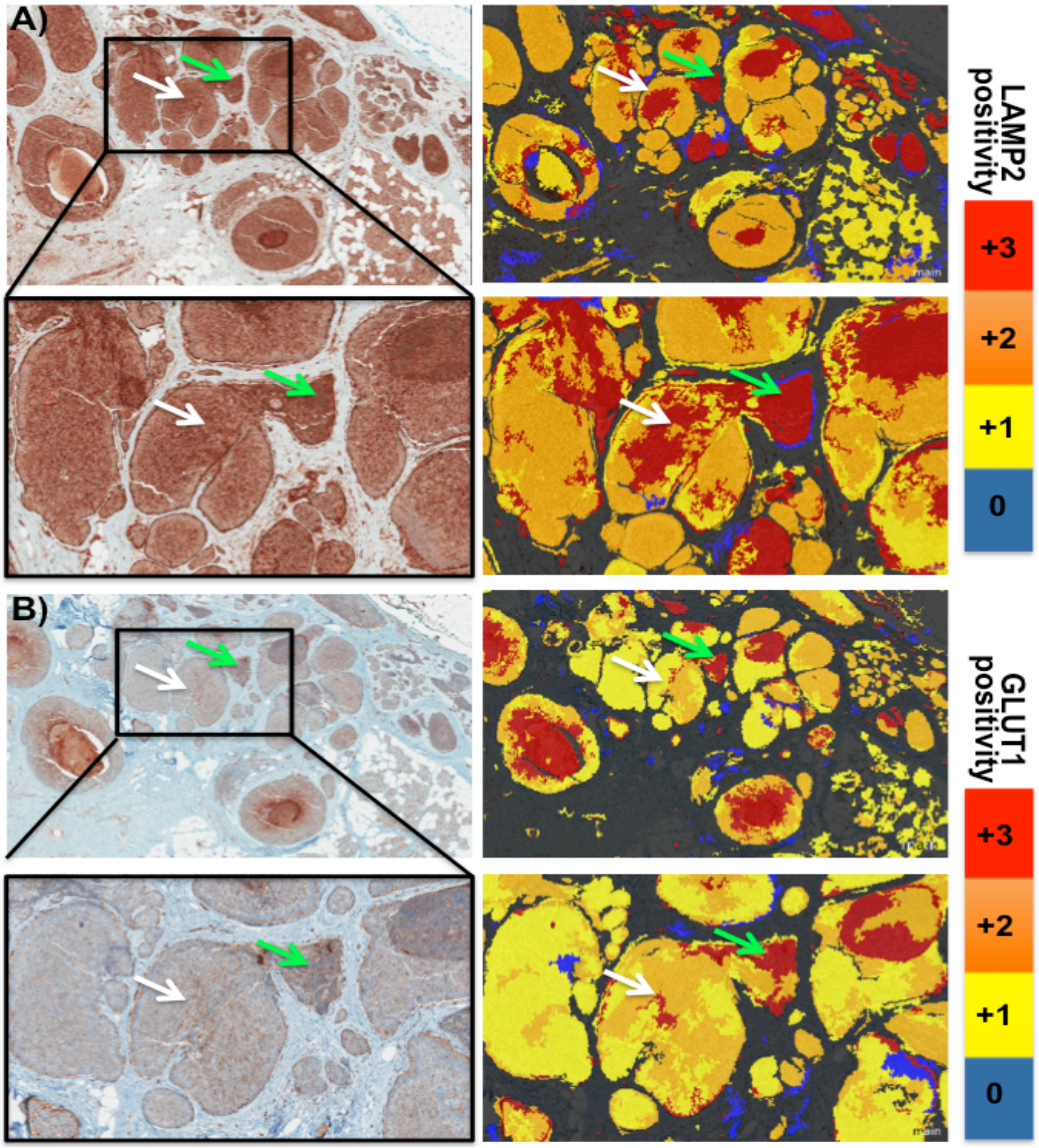

Co-registration of LAMP2 and Glut1 staining of whole tumor mount from patients. **A)** Left panel: IHC staining of a DCIS with LAMP2 antibody showed overexpression of this protein in regions expected to be more acidic such as centers of DCIS (white arrows) or DCIS with microinvasion (green arrows). Right panel: Color coded of the IHC section based on LAMP2 positivity (0-3). **B)** GLUT1 IHC staining of an adjacent section of the tumor used in A and pseudocolored presentation of its positivity (0-3). GLUT1 staining pattern is similar to the LAMP2 staining particularly in DCIS center.

Supplementary Figure 7.

A)

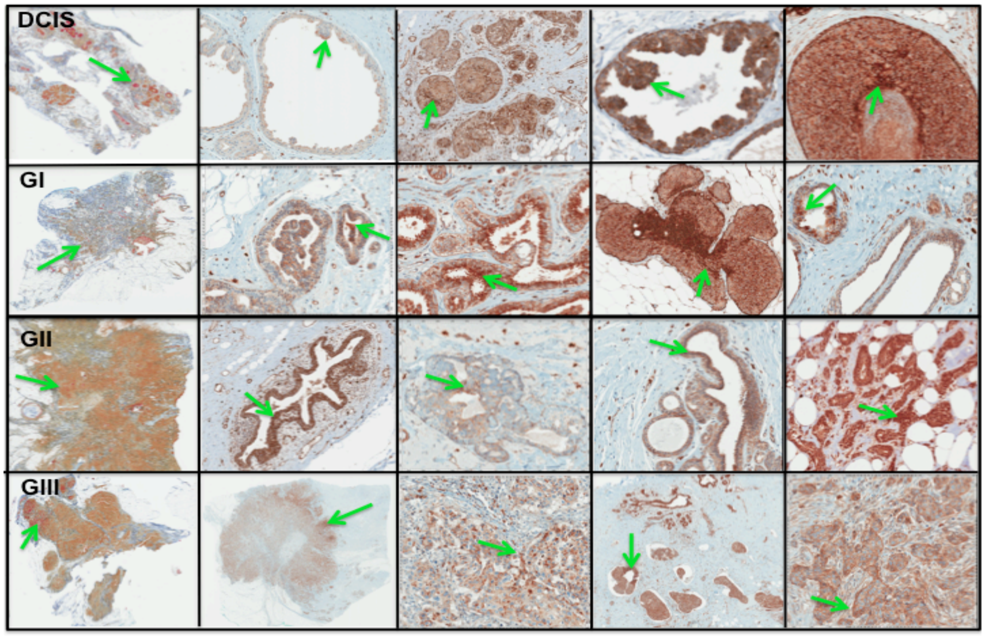

B)

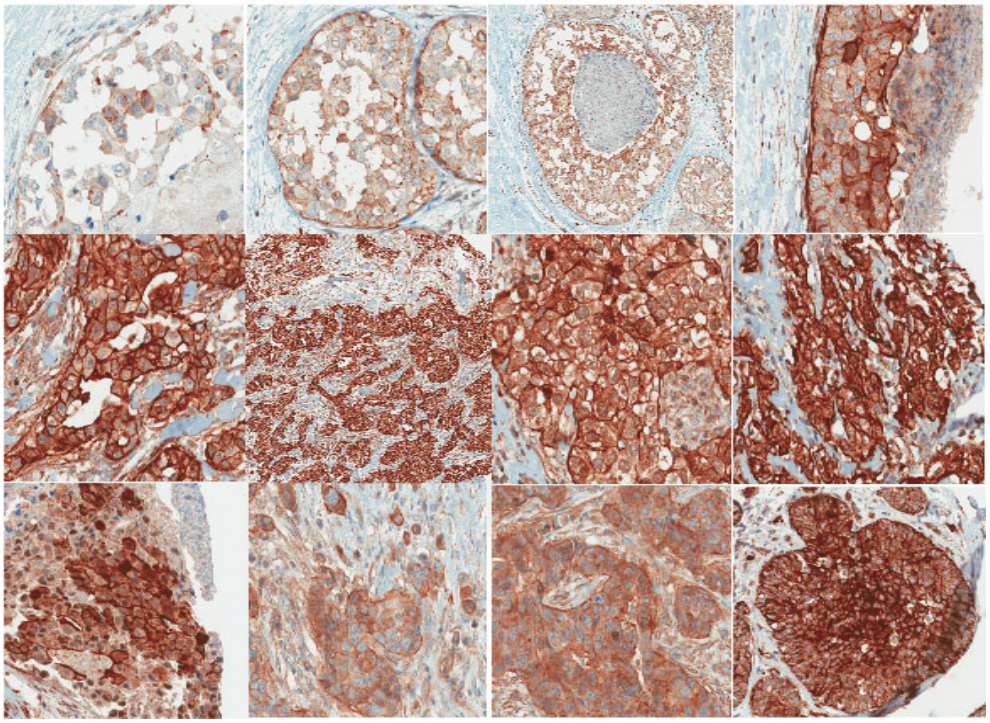

Overexpression of LAMP2 in acidic regions of breast cancer tumors. A) Representative LAMP2 IHC staining of whole mount tumor specimens. LAMP2 is over expressed at the regions that are expected to be acidic (green arrows). This can be used for future studies of designing imaging and therapeutic agents against acidosis in tumors and/or acid resistant cells. **B)** Overexpression of LAMP2 at the membrane of cancer cells in different stages of breast cancer tumors.

Supplementary Figure 8.

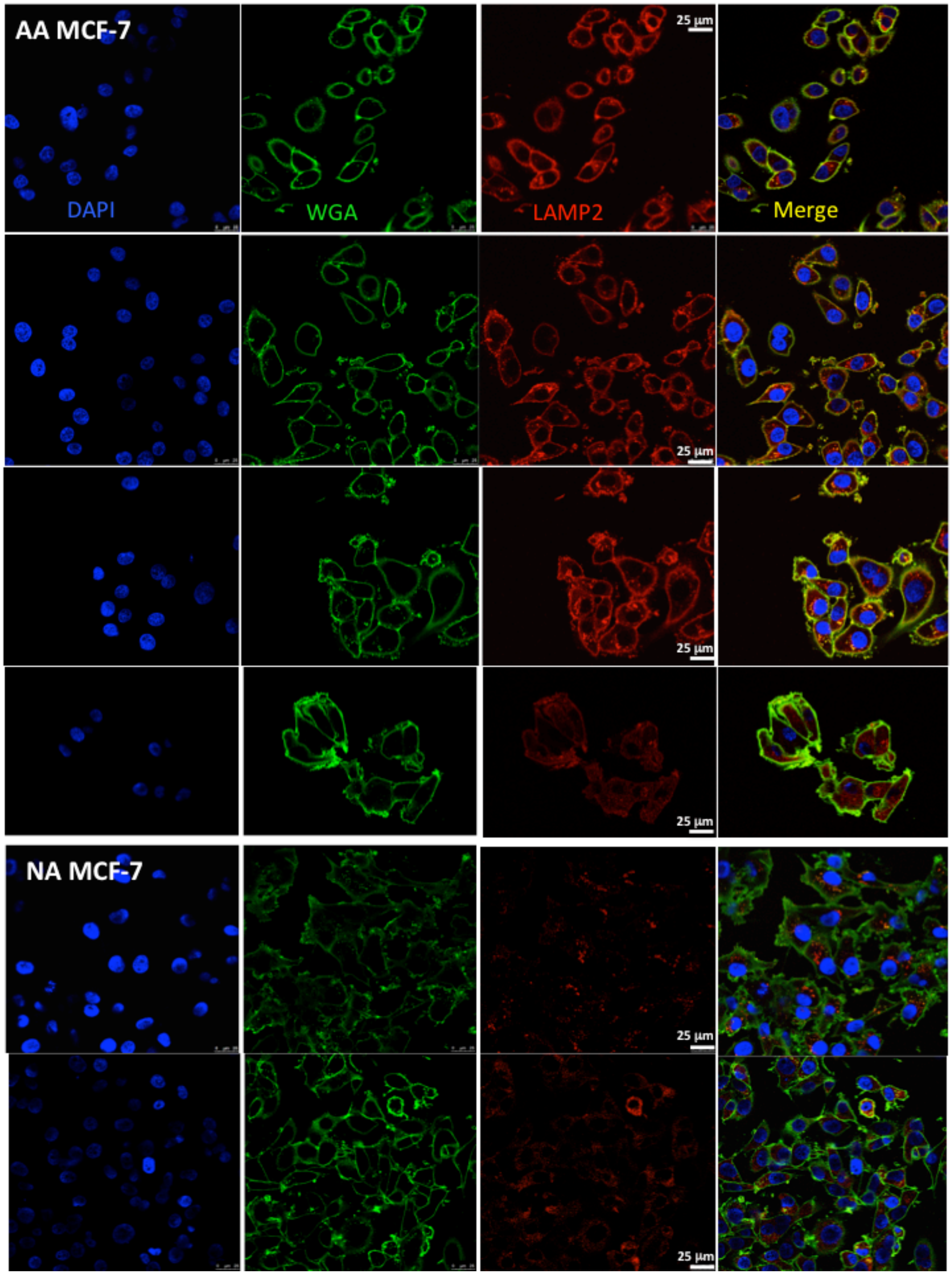

Representative images of LAMP2 immunocyto chemistry (ICC) staining in acid-adapted (top panel) and non-adapted (bottom panel) MCF-7 breast cancer cells. Staining with DAPI for nuclei (blue), LAMP2 antibody (red), Wheat Germ Agglutinin (WGA) for membrane (green), and merge images are shown. Acid-adapted cells had elevated LAMP2 staining at the cell-surface compared to non-adapted cells as indicated in the merged lower right image of each panel.

Supplementary Figure 9.

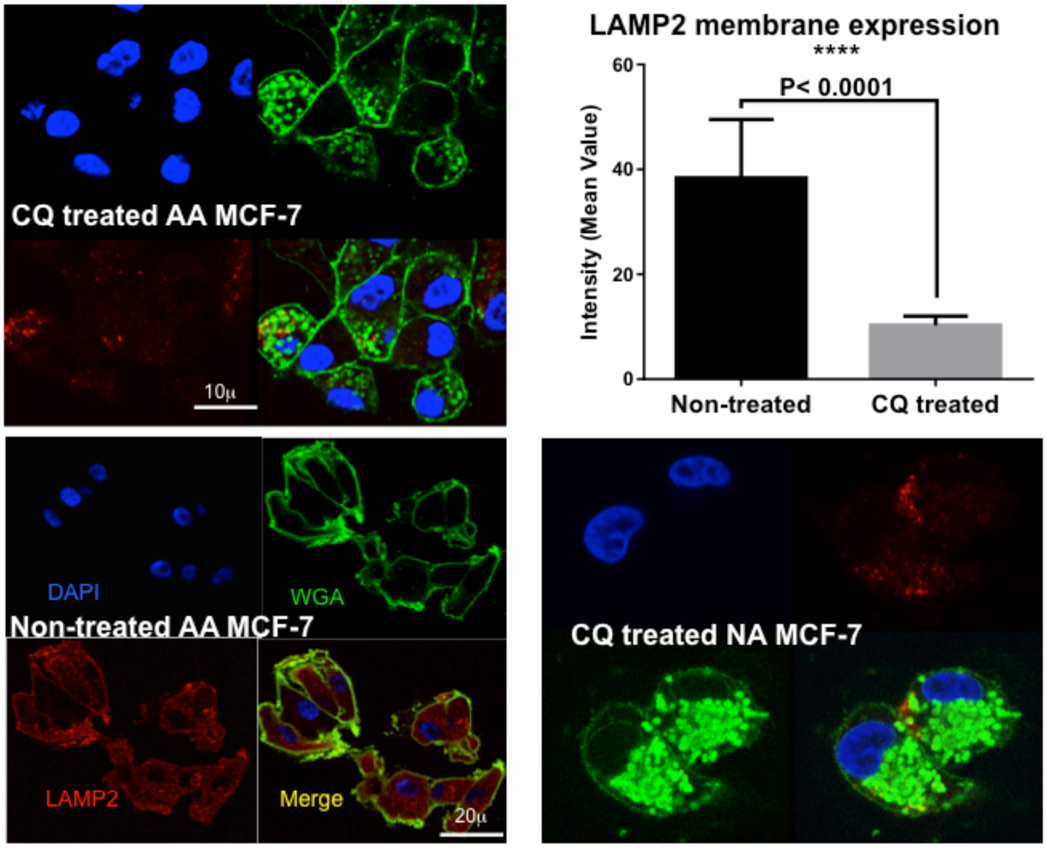

Immunocytochemistry of Chloroquine treated AA and NA MCF-7 cells and their analysis. Chloroquine reduces the plasma membrane expression of LAMP2.

Supplementary Figure 10.

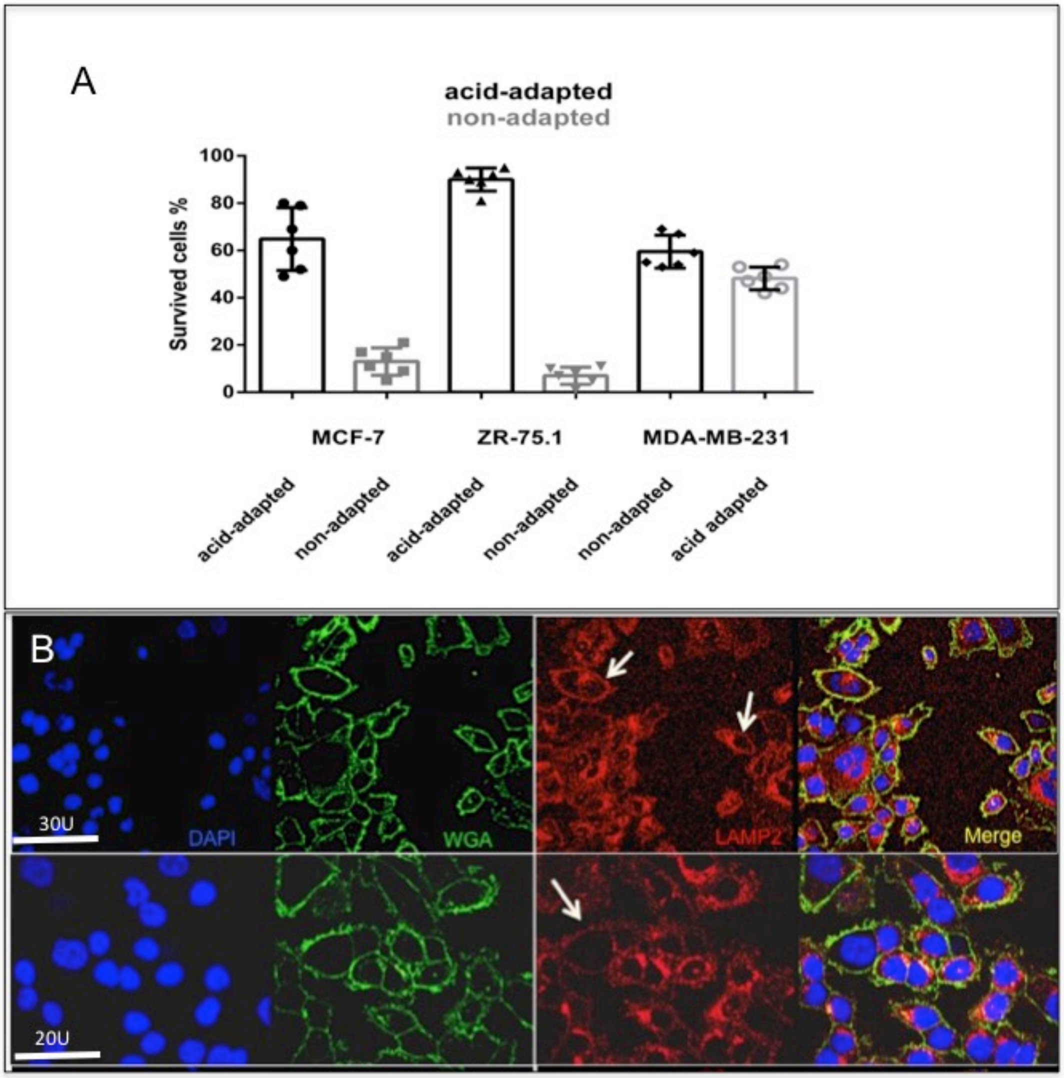

Acid sensitivity test of acid adapted and non-adapted cell lines. A) Survival assay of acid-adapted cells versus non-adapted ones in a media with pH 5.0 showed chronically acid-adapted cells tolerate acidic media much better than non-adapted cells. **B)** Immuno Cyto Chemistry of acid adapted versus non-adapted MDA-MB-231 cells shows LAMP2 expression at the cell surface of both groups.

Supplementary Figure 11.

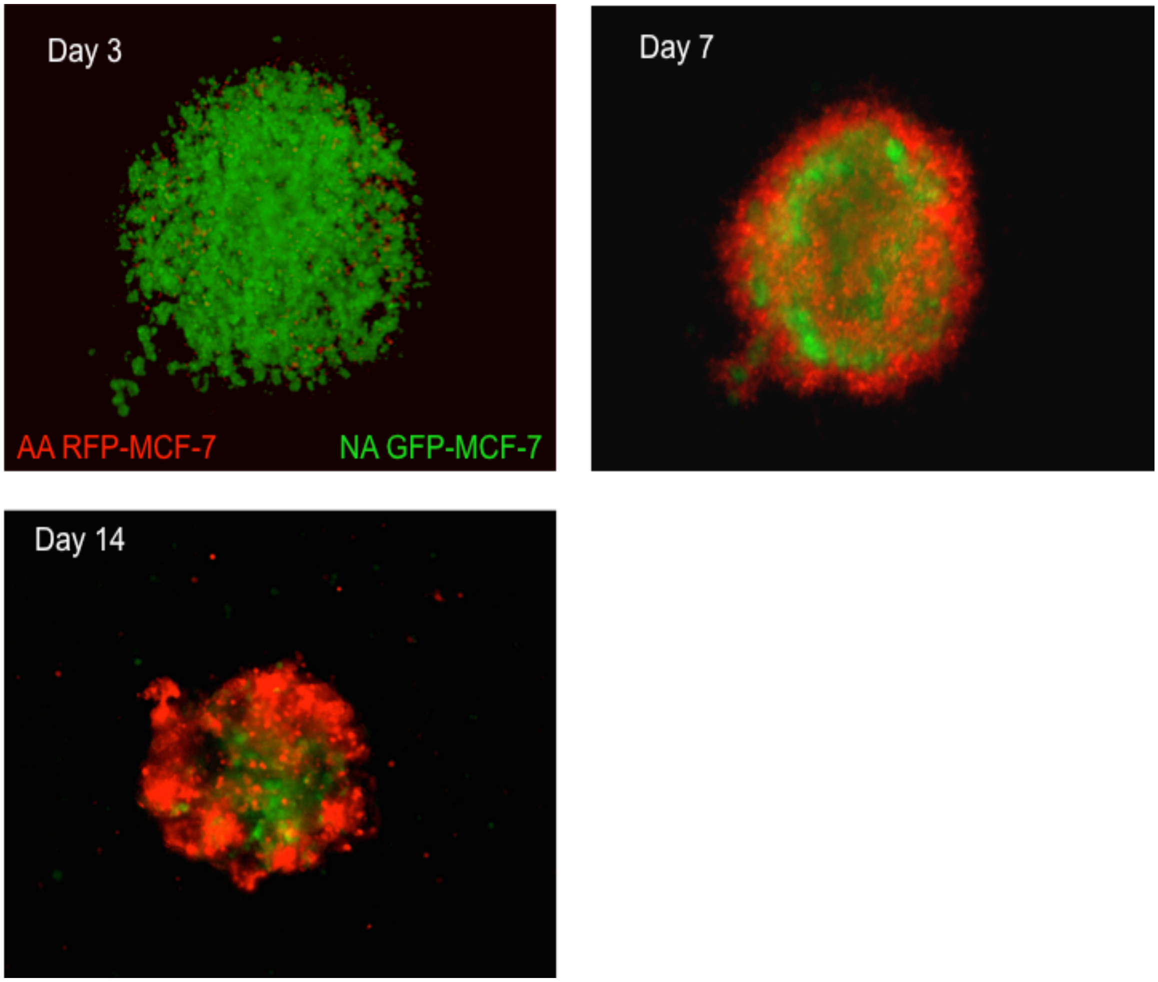

3D co-culture of AA and NA MCF-7 Cells. Co-culture of RFP-AA-MCF-7 cells with GFP-NA-MCF-7 cells as 3D spheroids (hanging drop) and growing them in low pH (6.5) revealed the resistance of acid adapted tumor cells and their over taking capability over non-evolved cells in a tumor like model.

Supplementary Figure 12.

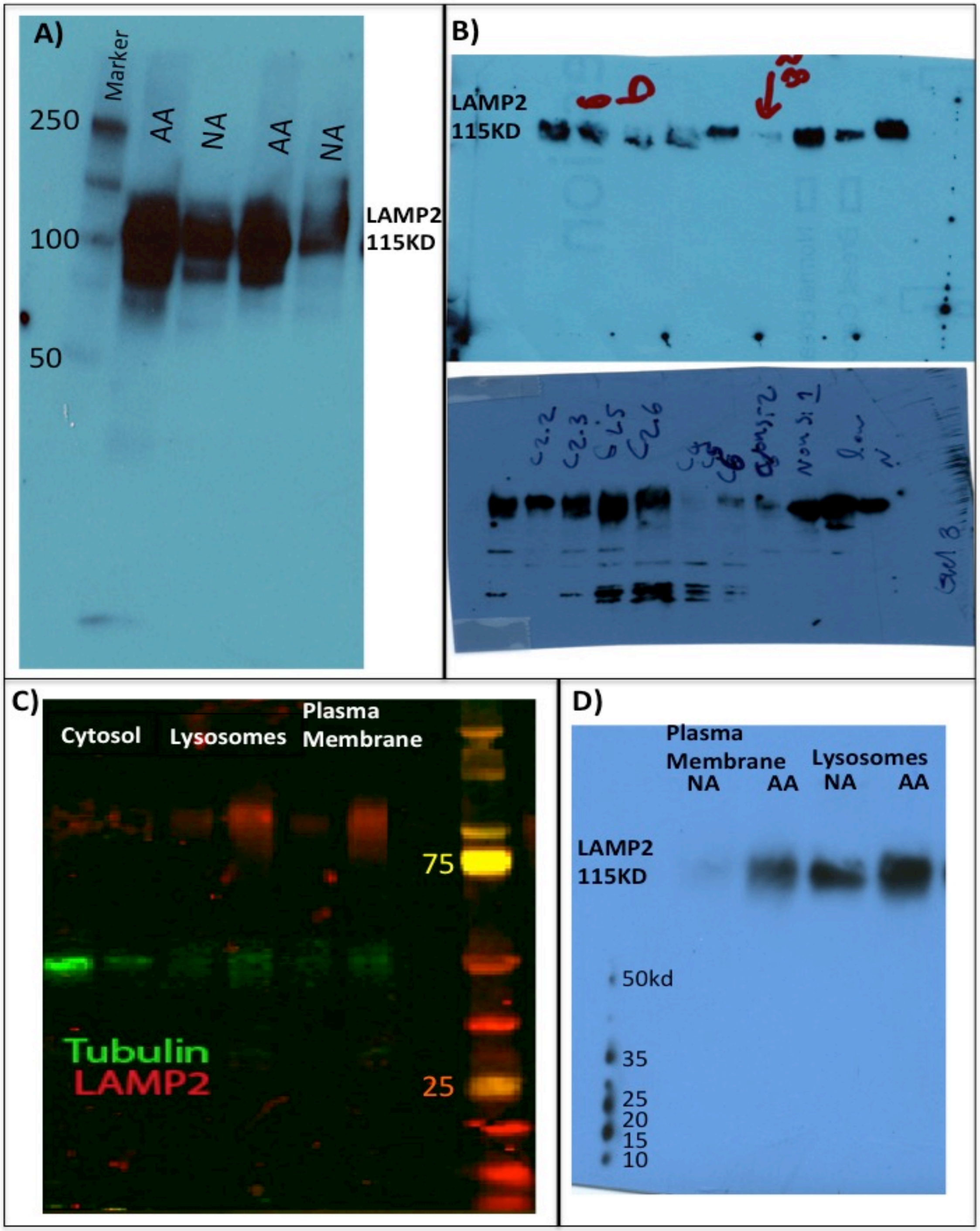

Full western blots that mentioned in figure 3 and 7 and supplementary figure 5.

Supplementary Table 1.

Protein ID	Gene ID	HL/LN	HN/LL
Four and a half LIM domains protein 1;Skeletal muscle LIM-protein 1	FHL1;SLIM1	17.366	0.56901
Protein-glutamine gamma-glutamyltransferase 2	TGM2	15.994	0.41472
Calcyclin;Growth factor-inducible protein 2A9;MLN 4;Protein S100-A6	S100A6	10.047	0.4073
Amplified and overexpressed in breast cancer	AIBC1;BCAS1	9.8043	0.37617
Differentiation-related gene 1 protein;Nickel-specific induction protein Cap43	CAP43;DRG1	9.516	0.34359
Heat shock-related 70 kDa protein 2	HSPA2	6.8706	0.57019
F-box only protein 20;LIM domain only protein 7;LOMP	FBX20;LMO7	6.1119	0.58144
Anoctamin-6;Transmembrane protein 16F	ANO6	5.4905	0.63386
F-box only protein 2	FBX2;FBXO2	5.3112	0.51243
CD49 antigen-like family member E;Fibronectin receptor subunit alpha;Integrin alpha-5	FNRA;ITGA5	5.1665	0.36882
Integral nuclear envelope inner membrane protein;Lamin-B receptor;LMN2R	LBR	5.097	0.61153
Aldo-keto reductase family 1 member C2;Chlordecone reductase homolog HAKRD	AKR1C2;DDH	4.6416	0.48092
Ephrin type-A receptor 2;Epithelial cell kinase;Tyrosine-protein kinase receptor ECK	ECK;EPHA2	4.5178	0.45831
Electroneutral potassium-chloride cotransporter 4;K-Cl cotransporter 4;Solute carrier family12 member 7	KCC4	4.0827	0.3788
Niemann-Pick C1 protein	NPC1	3.961	0.32178
APG9-like 1;Autophagy-related protein 9A	APG9L1	3.7837	0.39992
Growth/differentiation factor 15;Macrophage inhibitory cytokine 1;NSAID-activated gene 1 protein	GDF15;MIC1;PDF	3.6755	0.29873
Uncharacterized protein C10orf35	C10orf35	3.0925	0.33393
Amino acid transporter A2;Protein 40-9-1;Sodium-coupled neutral amino acid transporter 2	ATA2;KIAA1382	2.9642	0.29985
2-phospho-D-glycerate hydro-lyase;Enolase 2;Gamma-enolase;Neural enolase;Neuron-specific enolase	ENO2	2.9523	0.35578
Kinesin-like protein GAKIN;Kinesin-like protein KIF13B	GAKIN	2.737	0.33175
Ganglioside-induced differentiation-associated protein 2	GDAP2	2.6984	0.29846
GABA(A) receptor-associated protein-like 2;Gamma-aminobutyric acid receptor-associated protein-like 2	FLC3A;GABARAPL2	2.6192	0.40682
Phosphatidylinositol 4-kinase type 2-alpha;Phosphatidylinositol 4-kinase	PI4K2A	2.591	0.29549
UDP-N-acetylhexosamine pyrophosphorylase-like protein 1	UAP1L1	2.2829	0.38755
LMBR1 domain-containing protein 1	C6orf209;CD001	2.2455	0.31791
Membrane glycoprotein HP59;Sialin;Sodium/sialic acid cotransporter;Solute carrier family 17 member 5	SLC17A5	2.1884	0.24561
Niemann-Pick disease type C2 protein	NPC2	2.1348	0.4011
1-O-acylceramide synthase;Group XV phospholipase A2;Lysosomal phospholipase A2	LYPLA3;PLA2G15	1.9888	0.39956
Lysosome-associated membrane glycoprotein 2	LAMP2	1.9591	0.38888

List of the top 30 proteins discovered in SILAC proteomics with corresponding gene ID. The orange color provides results for one flipping experiment with acid-adapted MCF-7 cells (L) labeled with heavy (H) isotopes of Arginine and Lysine and non-adapted cells (N) with light isotope (L). Blue is the other experiment with the reverse labeling, low pH adapted labeled with light isotope (LL)/ normal pH labeled with heavy isotope (NH).
